# Phytosomal Delivery of Natural Bioactives: Advancing Bioavailability, Targeted Drug Delivery, and Clinical Translation

**DOI:** 10.3390/pharmaceutics18101232

**Published:** 2026-09-29

**Authors:** Mohammed Arfath Mysore Ismail, Akhilesh Dubey, Amitha Shetty, Haribalan Perumalsamy, Sri Renukadevi Balusamy, Seungah Lee, Manohar Mahadev

**Affiliations:** 1 Nitte (Deemed to be University), NGSM Institute of Pharmaceutical Sciences (NGSMIPS), Department of Pharmaceutics, Mangalore 575018, India; mohammed.24ppls104@student.nitte.edu.in (M.A.M.I.); akhilesh@nitte.edu.in (A.D.); amithashetty@nitte.edu.in (A.S.); 2 Center for Creative Convergence Education, Hanyang University, Seoul 04763, Republic of Korea; harijai2004@gmail.com; 3 Department of Chemistry, Hanyang University, Seoul 04763, Republic of Korea; 4 Department of Food Science and Biotechnology, Sejong University, Gwangjin-gu, Seoul 05006, Republic of Korea; renubalu@sejong.ac.kr; 5 Department of Applied Chemistry and Institute of Natural Sciences, Kyung Hee University, Yongin-si 17104, Gyeonggi-do, Republic of Korea; moon11311@naver.com

**Keywords:** natural bioactive compounds, phytosomes, phospholipid complexes, advanced drug delivery, bioavailability enhancement, phytopharmaceuticals

## Abstract

Natural bioactive compounds, especially plant-derived phytoconstituents, have gained significant interest due to their structural diversity, multitarget pharmacological activities, and favourable safety profiles. Their pharmaceutical translation is often hindered by poor aqueous solubility, limited membrane permeability, chemical instability, extensive first-pass metabolism, and, therefore, low oral bioavailability. Phytosomes, which are molecular complexes of bioactive compounds with phospholipids, have emerged as a new, effective, lipid-based delivery platform that can overcome these biopharmaceutical limitations and improve solubility, membrane interactions, systemic exposure, and therapeutic efficacy. The present review critically analyses recent advancements in phytosomal drug delivery, from formulation development to clinical translation, focusing on molecular complexation mechanisms, formulation strategies, physicochemical characterisation, pharmacokinetic enhancement, therapeutic applications, and challenges in clinical translation. The current review combines pharmaceutical design principles with recent advances in quality-by-design (QbD), computational formulation optimisation, hybrid phytosomal systems, scalable manufacturing approaches, and targeted delivery strategies. In addition, prospects for the future extension of phospholipid complexation to bioactive compounds from marine organisms, fungi, microorganisms, and algae are discussed in relevance to the next-generation natural product therapeutics. The present review serves as an emerging link between the discovery of natural bioactive compounds and the development of clinically translatable phytosomal systems from a pharmaceutical perspective.

## 1. Introduction

Natural bioactive compounds from medicinal plants and other biological sources have long served as therapeutic agents due to their remarkable structural diversity, multi-target pharmacological activities, and favourable safety profiles [1]. Phytochemicals such as flavonoids, polyphenols, alkaloids, terpenoids, saponins, and glycosides have been shown to exhibit significant therapeutic activity against various pathological disorders, including cancer, metabolic disorders, cardiovascular diseases, neurodegenerative disorders, inflammatory diseases, and microbial infections [2]. Many naturally occurring bioactive compounds are particularly attractive candidates for managing complex, multifactorial chronic diseases because they can exert pleiotropic pharmacological effects by modulating multiple cellular signalling pathways [3]. However, many phytoconstituents have unfavourable biopharmaceutical properties, such as low aqueous solubility, poor membrane permeability, chemical instability, rapid metabolic degradation, and extensive first-pass metabolism, which pose significant obstacles to their successful clinical translation. These limitations often result in low oral bioavailability, inadequate systemic exposure, and inconsistent therapeutic outcomes, thereby restricting their pharmaceutical and clinical applications [4]. To address these challenges, significant research has focused on the design and optimisation of advanced drug delivery systems to enhance the physicochemical stability, absorption, pharmacokinetic characteristics, and tissue distribution of natural bioactive molecules. In this regard, lipid-based nanocarriers have proven to be among the most effective delivery systems. The use of phospholipids to complex phytoconstituents is one of the most promising strategies to improve their therapeutic potential and translational relevance [5].

Although phytosomes are grouped with other lipid-based nanocarriers, including liposomes, solid lipid nanoparticles (SLNs), and nanostructured lipid carriers (NLCs), they differ from these systems with respect to their structural organisation and the modes of drug association (Figure 1) [6]. For instance, in liposomes, drugs are typically encapsulated within an aqueous core or embedded within the lipid bilayer [7]. Conversely, SLNs and NLCs utilise solid or hybrid lipid matrices to disperse drugs and achieve sustained release, particularly for lipophilic compounds. Phytosomes, however, form a defined molecular complex between the active constituent and the phospholipid [8], resulting in stable phyto-phospholipid complexes that synergistically optimise solubility, permeability, and, in some cases, targeted tissue distribution [9]. As a result, phytosome technology has become increasingly popular in the fields of nutraceutical, herbal, and pharmaceutical drug development.

Several reviews have addressed phytosome technology, emphasising its fundamental principles, bioavailability enhancement, formulation approaches, and therapeutic applications. The present review differs by integrating phytosome technology with a pharmaceutical and translational framework, linking recent advances in formulation and characterisation with reported physicochemical and biological characteristics, pharmacokinetic outcomes, therapeutic applications, and available clinical evidence. In particular, the formulation and characterisation literature is systematically synthesised using the PRISMA framework, with the selected dataset presented in Table 1, enabling recent preparation approaches and key formulation attributes to be considered alongside their reported performance. The review further brings together evidence on clinical progress and the practical challenges of standardisation, scalable manufacturing, regulatory aspects, and clinical validation. By connecting these aspects across the formulation-to-translation pathway, this review provides a focused assessment of the current development status of phytosomal drug delivery and identifies areas requiring further investigation for its continued pharmaceutical development.

## 2. Current Status of Phytosome Research

### 2.1. Search Strategy

A comprehensive literature search was conducted across Scopus, Web of Science, and PubMed for publications from 2020 to 2025 using the keywords “Phytosome” OR “Phytosomes” OR “Phyto-Phospholipid Complex” OR “Drug-Phospholipid Complex” OR “Phospholipid Complex” OR “Phosphatidylcholine Complex”. The search was performed over this broader period to identify relevant literature, while studies published between 2023 and 2025 were subsequently considered for inclusion according to the predefined inclusion criteria.

### 2.2. Inclusion Criteria

Articles were included if they were published in English, available in full text, and published between 2023 and 2025. Eligible studies were required to report original research on phytosome or phospholipid complex formulations, describe the preparation method, and provide relevant formulation and physicochemical characterisation data. Full-text accessibility was required to enable complete assessment of the reported information during screening and data extraction. The 2023–2025 restriction applied specifically to the systematic formulation and characterisation dataset presented in Table 1. Earlier publications were retained selectively as background, mechanistic, methodological, or historical references where necessary to contextualise the findings and were not included in the systematic dataset.

### 2.3. Exclusion Criteria

Articles were excluded if they were not published in English, were published outside the predefined 2023–2025 inclusion period, or were not available in full text. Reviews, abstracts, conference papers, retracted articles, animal-only studies, and other non-original research were excluded. Studies unrelated to phytosome or phospholipid complex technology or lacking sufficient information on formulation preparation or characterisation were also excluded.

### 2.4. Data Extraction & Quality Assessment

The exported results from Scopus, Web of Science, and PubMed were imported into Microsoft Excel, where duplicate records were identified and removed. Two reviewers independently screened titles and abstracts and subsequently assessed the full texts against the predefined inclusion and exclusion criteria; disagreements were resolved by a third reviewer. Data extraction was performed independently by two reviewers and cross-checked for accuracy. The methodological quality of the included studies was assessed using predefined criteria, with assessments performed independently by two reviewers and discrepancies resolved by discussion with a third reviewer.

### 2.5. Study Selection Process (PRISMA Flow)

The initial database search identified 895 records (Scopus: 196, Web of Science: 335, PubMed: 364). After removing 273 duplicates, 622 records were screened based on titles and abstracts; 285 were excluded according to the predefined criteria. Thus, 337 reports were selected for full-text assessment, of which 170 reports could not be retrieved. The remaining 167 reports were evaluated for eligibility, and 100 were excluded due to lack of online accessibility (n = 71), clinical studies without formulation or physicochemical characterisation data (n = 10), or insufficient formulation/characterisation details (n = 19). Finally, 67 studies met the inclusion criteria and were included in the qualitative synthesis. The study selection process is illustrated in the PRISMA flow diagram (Figure 2).

## 3. Mechanistic Basis of Phytosome-Mediated Drug Delivery

Phytosome formation arises from specific supramolecular interactions between natural bioactive compounds and phospholipids, in which noncovalent interactions yield stable amphiphilic complexes with distinct structural and biopharmaceutical properties, thereby transforming the bioactives’ physicochemical profile into an amphiphilic, membrane-compatible complex. Unlike conventional lipid carriers, this molecular association is stoichiometric and structurally organised, conferring predictable membrane-interaction behaviour and improved biological performance [11].

Fundamentally, phytosome formation begins with specific non-covalent interactions between bioactive compounds and phospholipid headgroups (Figure 3), mediated by hydrogen bonding and complemented by electrostatic interactions, π-π stacking, and van der Waals forces. These interactions occur between the polar functional groups of the bioactive compound, including phenolic hydroxyls, carboxylate groups, and carbonyl groups, and the polar head region of the phospholipid, especially the phosphate oxygens and the carbonyl groups in the glycerol backbone [12]. During these interactions, the phytoconstituent becomes molecularly attached to the zwitterionic phospholipid headgroup. At the same time, the hydrophobic fatty acyl chains remain mostly unaffected [13].

Following initial molecular anchoring, the phytoconstituent–phospholipid complexes undergo a secondary organisational process that leads to phytosome formation (Figure 3). This stage is characterised by supramolecular reorganisation and self-assembly of the stoichiometric molecular complexes in an aqueous environment. Upon hydration, the amphiphilic phytoconstituent-phospholipid adducts spontaneously rearrange to minimise interfacial free energy, giving rise to supramolecular assemblies in which the bioactive-phospholipid complex adopts an organised amphiphilic structure.

This molecular association and structural integration enhance membrane permeability and affinity by increasing lipophilicity and reducing crystallinity, thereby facilitating efficient partitioning into biological membranes. Since phosphatidylcholine is a major constituent of intestinal epithelial membranes, phytosome complexes exhibit biomimetic behaviour, facilitating closer contact, partial fusion, or transient integration with biological lipid bilayers, thereby enhancing membrane residence time and resulting in superior epithelial permeation and reduced efflux susceptibility compared to free bioactive compounds [11,14]. Additionally, the phospholipid component can provide a protective microenvironment for labile functional groups against harsh gastrointestinal conditions, thereby improving chemical and enzymatic stability [15,16]. These structural features collectively facilitate enhanced transcellular absorption and, in some cases, partial lymphatic uptake [17], thereby supporting the superior permeability, stability, and biopharmaceutical performance of phytosome-based drug delivery systems [11].

### 3.1. Role of Phosphatidylcholine as a Bioactive Carrier and Delivery Modulator

Phosphatidylcholine (PC) has a zwitterionic headgroup that supports hydrogen bonding and other polar interactions with a wide range of natural bioactive compounds, which helps to form stable molecular complexes. This particular structure contributes to colloidal stability and favours productive interactions with biological membranes [18,19]. At the molecular level, the stability of phytosomes is provided by the mutual interactions of natural bioactive compounds and phosphatidylcholine, in which the positively charged choline group can interact with both aromatic π-systems and negatively charged functions. In contrast, the phosphate group can act as a hydrogen-bond acceptor for phenolic hydroxyls. This combination of interactions provides stable stoichiometric complexes with enhanced functional performance [12].

In addition to its structural significance, PC’s biological compatibility enhances the efficacy of Phytosomes by providing naturally occurring components such as bile salts, lipoproteins, and cell membrane structures. As a result, it actively contributes to the human body’s lipid transport system, improving oral drug absorption by increasing micellar incorporation and lymphatic uptake, and reducing premature clearance [20].

### 3.2. Selection of Natural Bioactive Compounds for Phytosomal Drug Delivery

Phytosome technology offers an opportunity to apply natural bioactive compounds, which are generally polar to moderately amphipathic, have high pharmacological activity, but are limited by membrane permeability and oral bioavailability, and are classified as BCS class III or IV. The ability of these compounds to partition into lipids and cross membranes can be enhanced by forming a complex, while retaining their original chemical structure and pharmacodynamic properties [21].

Among the bioactive compounds, flavonoids (flavanols, flavones, isoflavones, and flavonolignans), ellagitannins, and phenolic glycosides are prominent examples that are particularly well suited to forming phytosomes due to the presence of many phenolic hydroxyl groups, which allow effective hydrogen bonding with the phospholipid head groups, thereby favouring the formation of supramolecular complexes [22]. Triterpenoid acids and certain anthraquinone derivatives are also capable of forming complexes, mainly through ionic or dipolar interactions with the choline group of phosphatidylcholines. Instead, highly lipophilic triterpenes, volatile monoterpenes, and high-molecular-weight polysaccharides are typically poor candidates because they lack sufficient polar functional groups, have steric limitations, or form complexes with low stability [23].

Thus, in general, the development of a phytosome depends on the right balance of molecular polarity, functional groups, and steric features. As such, the selection of natural bioactive compounds should be carefully and rationally determined to ensure the development of effective delivery systems [24].

While phytosome technology has been predominantly investigated for plant-derived phytoconstituents, its underlying phospholipid complexation mechanism is not inherently limited to botanical molecules. Bioactive compounds originating from marine organisms, microorganisms, fungi, algae, and other unconventional natural sources may also benefit from improved membrane permeability, stability, and systemic exposure when compatible molecular interactions with phospholipids can be achieved. Expanding phytosomal research to these emerging classes of natural bioactives represents a promising direction to broaden therapeutic applications and accelerate pharmaceutical translation.

## 4. Formulation Strategies Enabling Effective Phytosome Drug Delivery

### 4.1. Route 1: Solvent Evaporation-Based Complexation

The solvent evaporation method is the oldest and most conventional method used for the preparation of phytosomes, where the bioactive and phospholipids are dissolved in organic solvents like ethanol [19], or methanol, acetone, or dichloromethane [25], and then the solvent is evaporated under reflux (40–60 °C) to form the phospholipid complex. This technique is generally used to achieve high entrapment efficiency, but it can also result in relatively large particles. For instance, boswellic acid phytosomes developed for rheumatoid arthritis using Phospholipon^®^ 90H had a particle size of 441.12 ± 32 nm and an entrapment efficiency of 98.93 ± 0.78%, thereby improving bioavailability and anti-arthritic activity [26]. In the same way, halobetasol–phosphatidylcholine phytosomes obtained by refluxing and n-hexane precipitation were found to be suitable for topical psoriasis treatment, with a particle size of 200 ± 2 nm and 84% drug content. Size-reduction techniques are sometimes used in combination with solvent evaporation to form a nano-phytosome. For example, a pyrazoloquinolinone ligand phytosomal formulation for enhanced brain delivery was prepared via solvent evaporation and high-pressure homogenization, yielding 145–165 nm particles with entrapment efficiencies of 96.33–98.33% [19]. Similarly, the phytosomes of Hydnora abyssinica were prepared by solvent evaporation, homogenization, and ultrasonication, with a particle size of 72.56 ± 9 nm and an entrapment efficiency of 95.45 ± 3.20% [27]. Solvent evaporation thus proves to be a simple and powerful method for the synthesis of phytosomes. Meanwhile, its inherent limitation of larger particle size can be overcome by post-processing methods without affecting complex stability and entrapment efficiency.

### 4.2. Route 2: Thin-Film Hydration as a Delivery-Enabling Formulation Strategy

Phytosomes are prepared using the Bangham method (also known as the film hydration technique), which consists of two steps, wherein evaporation of the solvent in a controlled manner, followed by rehydration of the dry residue with phosphate-buffered saline (PBS) [28] or distilled water [29] or Normal saline (0.9% sodium chloride solution) or acetate buffer to form vesicular lipid complexes. This method usually yields multilamellar vesicles (MLVs), which can be converted into small unilamellar vesicles (SUVs) to improve pharmaceutical properties. For instance, the phytosomes of tetrahydrocurcumin for oral carcinoma were prepared using soy phosphatidylcholine, which resulted in a particle size of 317.35 ± 5.48 nm, a PDI of 0.285 ± 0.022, and a zeta potential of −29.88 ± 0.351 mV [29]. Likewise, Ampelopsis Radix phytosomes for colorectal cancer treatment had particle sizes of 85–130 nm, PDIs of 0.25–0.27, and zeta potentials of 12.26–18.70 mV, indicating better delivery properties [28]. However, in some cases, this technique is used in combination with probe sonication or homogenization. To reduce the particle size and achieve uniform dispersion, for example, hinokitiol phytosomes developed for breast cancer using thin-film hydration and high-speed homogenization exhibited particle sizes ranging from 138.4 ± 7.7 to 763.7 ± 15.4 nm, entrapment efficiencies of 29.16 ± 1.16–92.77 ± 7.01%, and zeta potentials of −10.2 ± 0.28 to −53.2 ± 1.06 mV [30]. Similarly, *Mesua ferrea* Linn. extract phytosomes prepared by the thin-film hydration and probe sonication method had a particle size of 72.16 ± 2.38 nm, a PDI of 0.22 ± 0.08, an EE of 60.71 ± 0.47%, and a zeta potential of 50.36 ± 0.43 mV [31]. In addition, rutin phytosomes prepared by a hybrid process comprising thin-film hydration, homogenization, and probe sonication had a particle size of 185 nm, a PDI of 0.392, an EE of 84.62%, and a zeta potential of −37.0 mV [32], demonstrating the effectiveness of hybrid processes in enhancing formulation performance. The studies together demonstrate the versatility of the thin-film hydration method and the role of post-processing methods in preparing phytosomes with improved stability and uniformity.

### 4.3. Route 3: Anti-Solvent Precipitation-Based Approaches (Including Co-Solvency)

The anti-solvent precipitation method is a quick, low-solvent, high-yielding process in which the bioactives and phospholipids are dissolved in a water-miscible organic solvent, such as dichloromethane [33], ethanol, methanol [34], or acetone, and then the mixture is refluxed to facilitate complexation. Then, a non-solvent or anti-solvent, such as n-hexane [35], water [36], or 2% acetic acid [37], can be used to precipitate the phospholipid complex. For example, tinospora cordifolia phytosome, prepared using n-hexane precipitation, showed a particle size of 76 μm, 95.91% entrapment efficiency, and 80% yield [34]. Likewise, a polyherbal phytosome containing Acalypha indica, Pergularia daemia, and Coleus amboinicus was prepared for pharyngitis, which resulted in a particle size of 158 nm, entrapment efficiency of 86.94 ± 0.3%, and percentage yield of 72.50%, indicating that it created a stable phytosome with favourable physicochemical characteristics [33]. These results indicate that using the anti-solvent precipitation method to prepare phytosomes can yield high EE and good physicochemical features.

The co-solvency method involves dissolving the bioactives and phospholipids in a compatible solvent system and then removing the solvent, typically by spray drying [38] or lyophilisation [39], to obtain a dry complex. This method is especially beneficial when the compound is only soluble in a few solvents. It has been extensively used to enhance the efficiency of complexation, stability, and bioavailability. For instance, a curcumin phytosomal complex with improved solubility, in vitro digestive and thermal stability was prepared using an ethanol-water (50:50) co-solvent system, homogenization, and spray drying, producing a particle size of 2.82 ± 0.19 to 26.42 ± 4.06 μm, entrapment efficiency of 86.50 ± 0.25–93.97 ± 0.27% and percentage yield of 70.01 ± 2.85–86.33 ± 3.51%, indicating the co-solvency approach is effective in producing a stable phytosomal complex with efficient drug incorporation and satisfactory production yield [38]. Likewise, the α-mangostin phytosomes for ovarian cancer therapy were prepared using the co-solvent system chloroform-methanol (10:1), complexed with Phospholipon^®^ 90G. Then, the solvent was evaporated, and the sample was lyophilised, yielding particles with a size of 138.9 nm, an entrapment efficiency of 95.10%, and a zeta potential of −44.5 ± 1.5 mV, indicating excellent colloidal stability and efficient incorporation of α-mangostin into the phytosomal matrix [39]. Moreover, a comparative study between co-solvency and thin-film hydration reported that the entrapment efficiency of the verbascoside-containing phytosomes (65.3 ± 2.1%) was significantly higher than that of verbascoside-loaded liposomes (30.83%) developed by the thin-film evaporation method. This improved incorporation efficiency highlights the potential of the co-solvency method to enhance the delivery and therapeutic activity of bioactive phytoconstituents [40]. In the same way, adventitious root phytosomes of Dendropanax morbifera treated with methyl jasmonate for anti-inflammatory applications were prepared by both the thin-film hydration and the co-solvency method, have reported entrapment efficiencies ranging from 43.14 ± 2.86% to 79.98 ± 1.45% and yields from 36.50 ± 3.64% to 69.17 ± 0.14% for the thin-film hydration method and entrapment efficiencies of 56.89 ± 3.09% to 71.19 ± 4.88% and yields of 51.71 ± 3.29% to 62.91 ± 3.11% for co-solvency method highlighting the selected technique can affect the formulations’ yield and the overall process performance [41]. These results also highlight the versatility of the co-solvency method for preparing phytosomes, which efficiently complexes phyto-phospholipids and is compatible with spray-drying and lyophilisation techniques, making it suitable for producing stable formulations for pharmaceutical and nutraceutical applications.

### 4.4. Route 4: Mechanical Dispersion and Emulsification Approaches (Solvent-Free or Low-Solvent Systems)

Mechanical dispersion and emulsification approaches are primarily employed for bioactives with limited intrinsic lipophilicity or high aqueous affinity. In these methods, bioactives are typically dispersed in an aqueous phase, often in the presence of surfactants and phospholipids dissolved or softened in a compatible organic or semi-organic medium [42]. Subsequent mechanical agitation, high-shear mixing, or emulsification promotes intimate contact between the phases, enabling the formation of a phyto-phospholipid complex. For example, a Pentaclethra macrophylla seed extract phytosomal formulation developed for anti-inflammatory applications utilised Phospholipon^®^ 90 H as the phospholipid and Kolliphor^®^ HS 15 as the surfactant, exhibited an entrapment efficiency of 92.99 ± 0.09% and a yield of 33.43%, indicating efficient incorporation of the bioactive constituents and supporting its potential as an effective phytosomal delivery system [43]. Similarly, Callistemon citrinus extract phytosomes developed for the management of obesity were formulated using soybean phosphatidylcholine, along with Tween 80 and ethyl acetate to enhance the solubility and emulsification of the system to yield a particle size of 129.98 ± 18.30 nm and an entrapment efficiency of 80.49 ± 0.07%, indicating successful incorporation of the bioactive constituents and highlighting the potential of phytosomal delivery to improve the therapeutic performance of the extract [44]. Therefore, mechanical dispersion is the best-suited method for hydrophilic bioactives to achieve high entrapment and practical yields via surfactant-assisted emulsification, thereby overcoming aqueous drug solubility limitations through lipid complexation and enhancing bioavailability.

### 4.5. Advanced Post-Processing Steps in Phytosome Preparation

The rotary evaporation and post-processing steps are important supporting steps in the preparation of phytosomes and are not independent fabrication steps. Rotary evaporation is primarily used for solvent removal and thin-film formation in Routes 1 and 2 [45]. In this process, reduced pressure and rotational motion help form a uniform lipid-drug film on the inner surface of the flask, which serves as a precursor to the hydration or precipitation step in complex formation [46].

After the primary fabrication process, optional post-processing methods such as high-pressure homogenization, ultrasonication, or mechanical grinding can be used to further refine particle size, improve dispersion uniformity, and enhance dispersion stability. Though they are not specific preparation techniques, they are important for preparing nanoscale particles from bulk phytosome complexes without affecting the efficiency of complex formation and entrapment [31,47]. Therefore, critical quality attributes such as particle size, polydispersity index (PDI), and dispersion stability should be considered emergent properties of the primary fabrication route and subsequent refinement processes.

### 4.6. Novel Formulation Approaches

Phytosome technology has evolved beyond conventional phospholipid complexes toward multifunctional formulations designed to address specific biopharmaceutical and therapeutic challenges. Recent innovations integrate surface engineering, hybrid carrier systems, and advanced manufacturing technologies to improve stability, bioavailability, tissue targeting, and therapeutic efficacy. These developments have transformed phytosomes from passive drug carriers into application-specific delivery platforms. For instance, selenized tripterine phytosomes for acute kidney injury had a particle size of ~200 nm and a zeta potential of −38.5 mV, demonstrating good colloidal stability and increased antioxidant activity and ferroptosis inhibition in the kidneys [48], representing an example of therapeutic component engineering, where selenium contributes additional antioxidant activity while enhancing the biological performance of the encapsulated compound. Likewise, hybrid phytosomal systems have also attracted considerable attention. For example, a neohesperidin phospholipid nanosystem, which combined phospholipid complexation with cyclodextrin inclusion to overcome poor aqueous solubility and oral bioavailability, yielded nanoparticles with particle sizes of 96.7–132.2 nm, PDI < 0.2, and entrapment efficiencies of 92.3–95.6%, which showed enhanced solubility, bioaccessibility, and antioxidant activity compared to free neohesperidin [49]. Similarly, hyaluronic acid-coated PEGylated fisetin cholephytosomes integrated PEGylation with hyaluronic acid-mediated surface functionalization to improve circulation time and cellular targeting, resulting in sustained drug release and nearly ten-fold greater cytotoxicity than free fisetin with particle size ranging from 226.67 to 277.76 nm, PDI of 0.19–0.44 and tunable surface charge of −21.73 to +51.43 mV [50]. Nevertheless, innovation has also extended to manufacturing technologies. For example, curcumin phytosomes prepared using a microfluidic, continuous-flow technique had a particle size of 71.19 ± 1.37 nm, a PDI of 0.226 ± 0.047, and a zeta potential of −38.23 ± 0.89 mV, resulting in markedly improved oral bioavailability and systemic exposure compared with conventional curcumin formulations [51]. Therefore, recent technological developments have greatly broadened the scope of phytosomal systems, allowing the formulation of improved systems and achieving better therapeutic results. These innovations are expected to shape the future of more effective, clinically relevant phytosome-based delivery platforms.

### 4.7. Methodological Trends in Phytosome Preparation

To put these methods into perspective, Figure 4 illustrates the different methods used to prepare phytosome-based formulations. Among the different approaches, the majority rely on thin-film hydration (Route 2), accounting for approximately 42% of all approaches due to its stability, reproducibility, and broad applicability to various phytoconstituents. In addition, mechanical dispersion-based approaches (Route 4, approximately 16%) and solvent-based evaporation techniques (Route 1, approximately 15%) have also been widely used owing to their ease of use, scalability, and compatibility with existing pharmaceutical manufacturing techniques. In some instances, mechanical dispersion techniques have been used as supplementary methods to improve particle uniformity. The anti-solvent precipitation approaches (including co-solvency) (Route 3, approximately 19%) are not commonly used and are usually applied in more complex formulation processes. A smaller group of studies (8%) focuses on newer or hybrid approaches such as supercritical fluid processing, microwave-assisted complexation, or spray-drying-assisted approaches. The limited adoption of these methods reflects ongoing optimisation and the current lack of standardisation for routine phytosome production.

## 5. Key Characterisation Attributes of Phytosome Systems for Delivery Applications

Comparative analysis of the studies compiled in Table 1 highlights considerable variation in the reported physicochemical characteristics of phytosomal formulations. These differences across bioactive compounds, formulation compositions, and preparation approaches indicate that particle size, PDI, entrapment efficiency, and zeta potential are formulation-dependent rather than characterised by a single profile. Such differences in formulation characteristics can also be reflected in formulation performance, including drug-release behaviour. These comparative observations are further examined in the following sections by considering particle size, PDI, zeta potential, entrapment efficiency, drug loading, and yield in relation to formulation composition, processing, and delivery requirements.

### 5.1. Particle Size Distribution and Polydispersity Index (PDI)

Particle size and the polydispersity index (PDI) are the most important quality attributes of phytosomes, which determine colloidal stability, cellular uptake, and biodistribution. When examining the reported studies, it is evident that there is significant variation in these parameters, which depends on the therapeutic goals and formulation approaches used. For example, the ferulic acid phytosomes targeted to the liver had a particle size of 3.08 ± 0.01 nm and demonstrated high bioavailability, improved tissue retention, and emphasised the importance of nanoscale dimensions for targeted delivery [52]. Likewise, *Mesua ferrea* Linn. phytosomes formulated for atopic dermatitis had a particle size of 72.16 ± 2.38 nm and a PDI of 0.22 ± 0.08, which enhanced skin permeation and therapeutic activity [31].

Larger systems, such as boswellic acid phytosomes (441.12 ± 32 nm), have been successfully used to treat rheumatoid arthritis [26], demonstrating improved bioavailability and anti-inflammatory effects. The wide size range reported in the literature, from 3.08 nm to 76 μm [53], indicates that no universal optimum size exists. Similarly, low PDI values (<0.3) were found for ferulic acid phytosomes (0.101 ± 0.02) [52] and pomegranate phytosomes (0.168) [54], indicating uniform particle populations suitable for repeatable delivery. On the other hand, higher PDI values may indicate greater heterogeneity and reduced colloidal stability [37]. The different particle sizes and PDI reported so far are shown in Table 1. These combined results show that particle size and PDI should be adjusted based on therapeutic use and route of administration, with formulation composition, drug-to-phospholipid ratio, and post-processing techniques being important for achieving the desired particle size and PDI.

### 5.2. Zeta Potential and Surface Charge Behaviour

The zeta potential (ZP) is one of the most important quality parameters of phytosomal systems, as it can affect their stability, particle aggregation, cell interactions, and drug-release behaviour. Different formulations and applications often yield different reported zeta potentials. For instance, Rubia cordifolia phytosomes formulated for neuropathic pain had a relatively low ZP of −6.92 ± 0.10 mV [55]. In contrast, the ZP of diosmetin phytosomes designed to facilitate oral delivery was high at −115.1 mV [56], indicating strong electrostatic stabilisation. Likewise, phytosomes of α-mangostin and pomegranate for ovarian cancer and antioxidant applications, respectively, had a ZP of −44.5 ± 1.5 mV [39] and −40.15 mV [54]. On the contrary, positively charged systems have been explored to enhance interactions with negatively charged cellular membranes or specific negatively charged biological interfaces, such as *Mesua ferrea* Linn. phytosomes for atopic dermatitis (+50.36 ± 0.43 mV) [31] and Mastoparan-M phytosomes for breast cancer (+32.05 ± 17.27 mV) [57]. The zeta potentials reported so far are represented in Table 1. Recently, fisetin-loaded cholephytosomes designed for breast cancer treatment, with added stearylamine, TPGS, and hyaluronic acid, have enhanced zeta potential, cell interaction, targeting, and anticancer effects, shifting from −21.73 mV to +51.43 mV [50]. emphasising the growing importance of surface engineering in imparting the desired therapeutic properties to phytosomal systems. As a result, zeta potential has become an important design factor that affects the stability of colloids and the biological fate and therapeutic effects of phytosomal formulations.

### 5.3. Encapsulation Efficiency, Drug Loading, and Percentage Yield

The key formulation parameters for assessing the efficiency of bioactive compound incorporation and delivery in phytosomes are encapsulation efficiency (EE), drug loading (DL), and percentage yield (%Y). Reported studies show significant differences in these parameters that depend on the physicochemical properties of the bioactive and the formulation strategy used. For instance, geraniin phytosomes with an entrapment efficiency of 100.2 ± 0.8% and a significant enhancement in pharmacokinetic performance were developed to improve oral bioavailability [58]. Similarly, neohesperidin phospholipid-cyclodextrin nanosystems for enhancing its bioavailability and entrapment efficiency ranged from 92.3 ± 1.2% to 95.6 ± 1.2%, thereby improving its bioaccessibility and antioxidant activity [49]. The loading capacity of a drug also varies significantly depending on its formulation composition. For instance, curcumin phytosomes prepared with phosphatidylcholine to increase formulation solubility and thermal stability achieved a high drug loading of up to 52% [42].

In contrast, *Mesua ferrea* Linn. phytosomes prepared with soya lecithin and cholesterol showed a DL of 6.93 ± 0.33% [31], indicating that formulation composition and drug–phospholipid interactions can influence drug-loading capacity. These results suggest that developing phytosomal systems optimally involves not just maximising EE, DL, or %Y, but also attaining the best values of these parameters suited to a particular therapeutic purpose. Nevertheless, customised loading capacities and process efficiencies could be advantageous for targeted delivery systems to support specific clinical objectives.

### 5.4. In Vitro Release Profiles: Drug Kinetics and Delivery Mechanisms

In vitro release studies are essential for understanding how phytosomal systems regulate the release of encapsulated bioactive compounds and how these release characteristics influence their therapeutic performance. Unlike free bioactives, which often suffer from poor aqueous solubility, limited permeability, rapid degradation, and low bioavailability, phytosomes form stable phospholipid complexes that enhance drug stability and modulate the release profile. Consequently, phytosomal encapsulation not only enhances bioavailability but also prolongs the availability of bioactives at the site of absorption or action, thereby improving therapeutic efficacy. Across the reported studies, phytosomal formulations consistently exhibited release profiles that differed from those of their corresponding free compounds, primarily due to phospholipid complexation, which affected drug diffusion, matrix retention, and bioactive stability. For example, quercetin phytosomes designed to improve oral bioavailability achieved a cumulative drug release of 98.1 ± 6.59% after 480 min compared with 68.6 ± 7.6% for free quercetin under the same conditions [59]. Similarly, although free naltrexone hydrochloride was completely released within 8 h, its phytosomal formulation exhibited a sustained-release profile, releasing only 68.2 ± 1.5% of the drug over 72 h, thereby extending drug exposure and potentially reducing dosing frequency [60]. The observed differences in drug release are governed by several formulation-related factors rather than a single parameter. Particle size, entrapment efficiency (EE), drug loading, phospholipid composition, and the strength of drug-phospholipid interactions collectively determine the release behaviour of phytosomal systems. In general, smaller phytosomes provide a larger surface area for drug diffusion, which may accelerate release. In contrast, stronger drug entrapment within the phospholipid matrix generally promotes sustained drug release. For example, Ampelopsis Radix phytosomes with particle sizes of 85–130 nm exhibited controlled release suitable for prolonged therapeutic action [28].

In contrast, boswellic acid phytosomes prepared for the treatment of rheumatoid arthritis exhibited a larger particle size (441.12 ± 32 nm) and a higher entrapment efficiency (98.93 ± 0.78%), resulting in greater drug retention within the lipid matrix, gradual drug release, enhanced bioavailability, and improved anti-inflammatory activity [26]. The complexity of phytosomal drug release is further reflected in the kinetic models used to describe the release mechanism. Depending on formulation composition and drug-phospholipid interactions, phytosomal systems may follow diffusion-controlled, anomalous transport, or concentration-dependent release pathways.

**Table 1:** Summary of phytosome-based formulations reported in recent research articles.

Sl. No	Drug/Extract	Pharmacological Activity/Intention	Phytosome Composition	Administration	Characterisation PS (nm) PDI EE (%) ZP (mV)	Inference	Reference
Solvent evaporation method							
1	*Aegle marmelos* extract	Ulcerative colitis	*Aegle marmelos* extract + phosphatidylcholine	NR	PS- 673.6 ± 4.30 PDI- 0.224 ± 0.010 EE- 83.15 ± 1.04 ZP- −42.6 ±0.51	Phospholipid complexation significantly increased the antioxidant and anti-inflammatory activities, demonstrating the formulation’s increased efficacy and therapeutic potential compared to the simple extract.	[61]
2	Halobetasol	Psoriasis	Halobetasol + phosphatidylcholine	Topical	PS- 200 ± 2.0 PDI- 0.224 ± 0.05	Halobetasol–phosphatidylcholine complex gel successfully reduces psoriatic symptoms when applied topically, suggesting increased treatment efficacy.	[20]
3	Cannabidiol	To Improve Water Solubility and Permeability	Cannabidiol + L-α-Phosphatidylcholine from dried egg yolk	Oral	At pH 6.8 PS- 215.9 PDI- 0.3 At pH 7.4 PS- 600.5 PDI- 0.4 EE- 78.53 ± 6.40	The cannabidiol-phospholipid complex greatly improved stability, permeability, and solubility, indicating a promising strategy to overcome limitations of oral delivery.	[62]
4	Boswellic acids	Rheumatoid arthritis	Boswellic acids + Phospholipon^®^ 90H	Oral	PS- 441.12 ± 32.0 EE- 98.93 ± 0.78 ZP- − 36.35 ± 1.19	Boswellic acid naturosomes significantly improved solubility, stability, and bioavailability, resulting in greater anti-arthritic efficacy than the plain extract.	[26]
5	Naltrexone hydrochloride	To improve Drug loading	Naltrexone hydrochloride + Soya phosphatidylcholine + Cholesterol + triolein, L-lysine, sucrose, dihexacetyl phosphate (DCP)	NR	PS- 10.2 ± 0.2 µm EE- 19.8 ± 0.5	Naltrexone hydrochloride–phospholipid complexation enhanced lipophilicity, encapsulation efficiency, and sustained drug release in MVLs, highlighting its potential as an effective controlled-release delivery system.	[60]
6	Ferulic acid	Liver targeting	Drug + LIPOID^®^SPC-3 + Galactose (GTE)	Intranasal	PS- 3.08 ± 0.01 PDI- 0.01 ± 0.02 ZP- −12.15	Ferulic acid phytosomal soft nanoparticles increased the solubility, bioavailability, and retention of ferulic acid, indicating a high potential for targeted treatment of liver cancer.	[52]
Solvent evaporation + High-pressure homogenization							
7	Pyrazoloquinolinone ligand (CW-02-79)	To Enhance Brain Delivery	Pyrazoloquinolinone ligand CW-02-79 + soy lecithin	Intravenous	PS- 145 to 165 PDI- < 0.25 EE- 96.33 to 98.33 ZP- –37.5 to –48	A phospholipid complex-based nanoemulsion increased CW-02-79’s solubility, stability, and brain-targeting efficiency, demonstrating its promise as a biocompatible approach for enhanced brain delivery.	[19]
8	Aprepitant	To prepare an intravenous emulsion loaded with phytocomplex	Aprepitant + Egg yolk lecithin	Intravenous	PS- 82.83 ± 1.89 PDI- 0.243 ± 0.008 EE- 98.84 ± 1.43 ZP- −59.0 ± 2.54	An Aprepitant-phospholipid complex emulsion demonstrated improved stability, safety, bioavailability, and liver-targeting efficacy, indicating a high potential for intravenous administration.	[63]
Solvent evaporation + Homogenization + Ultrasonication							
9	*Hydnora abyssinica*	To improve the Functionality of yoghurt by adding the phytocomplex for its prebiotic and antimicrobial activity	*Hydnora abyssinica* + phosphatidylcholine	Oral	PS- 251 ± 37 to 380 ± 56 PDI- 0.283 to 0.398 EE- 90.68 ± 3.27 to 95.45 ± 3.20 ZP- −29.5 ± 0.19 to −37 ± 0.05	Hydnora abyssinica phytosomal-supplemented yoghurt improved antioxidant activity, texture, shelf life, and acceptance, demonstrating high potential as a functional dairy product.	[27]
Solvent evaporation method + Probe sonication							
10	*Cyclopia subternata* extract	To understand the effect of process conditions and stability assessment	*Cyclopia subternata* extract + Lipoid S 40	NR	PS- 113.7 to 312.7 PDI- 0.31 to 0.48 EE- 3.7 to 79.0 ZP- −55.0 to −38.9	Nano-phytosomes improved stability and protection of phenolic compounds, indicating potential for functional food applications.	[64]
Rotary Evaporation/Thin-film formation method							
11	Quercetin	anti-obesity	Quercetin + L-α phosphatidylcholine	Oral	PS- 204.6 PDI- 0.45 EE- 84.48 to 88.59 ZP- −28.6	Optimised quercetin phytosomes improved stability and drug delivery performance, indicating potential as a safe anti-obesity agent, though further clinical validation is required.	[59]
12	*Milk thistle* + Curcumin extract + *Ginkgo biloba* extract	PCOD	Poly herbal + soy lecithin	NR	EE- 65.91 ZP- −29.6	Phytosomes improved bioavailability and therapeutic efficacy of herbal bioactives, addressing key PCOS-related conditions.	[65]
13	Vitexin	Antioxidant	Vitexin + PC from soybean (SPC) or egg yolk (EPC)	NR	In SPC PS- 468.20 ± 31.40 PDI- 0.47 ± 0.02 EE- 98.19 ± 0.05 ZP- −37.10 ± 3.52 In EPC PS- 290.93 ± 11.89 PDI- 0.46 ± 0.03 EE- 98.27 ± 0.03 ZP- −37.00 ± 3.29	Vitexin phytosomes enhanced solubility, stability, antioxidant activity, and bioaccessibility; further safety evaluation is required.	[66]
14	Gentiopicroside	Type 2 diabetes	Gentiopicroside + Soybean phospholipids	Oral	In 0.1N HCL PS- 25.2 ± 5.3 ZP- −19.23 ± 5.84 In PBS pH 6.8 PS- 46.7 ± 8.2 ZP- −23.43 ± 3.93	With higher Cmax and AUC, the Gentiopicroside-phospholipid complex-loaded SNEDDS improved bioavailability (2.6- and 1.3-fold increases over the suspension and oil solution, respectively), suggesting effective oral administration.	[67]
Thin-film hydration method							
15	Tetrahydrocurcumin	Oral carcinoma	Tetrahydrocurcumin + soy phosphatidylcholine	NR	PS- 317.35 ± 5.48 PDI- 0.285 ± 0.022 ZP- −29.88 ± 0.351	THC-phytosomes were more effective than conventional chemotherapy and showed great promise as a targeted anticancer delivery strategy.	[29]
16	*Ampelopsis Radix*	Colorectal cancer	*Ampelopsis Radix* + Egg phosphatidylcholine (EPC) + Cholesterol	NR	PS- 85 to 130 PDI- 0.25 to 0.27 ZP- 12.26 ± 1.3 to 18.70 ± 2.5	Phytosomes loaded with Ampelopsis Radix extract exhibited improved properties, increased absorption, and superior anticancer activity, with 100% survival, suggesting great promise for treating colorectal cancer.	[28]
17	*Gymnema inodorum* extract	To Prevent Inflammation in Macrophages and Insulin Resistance in Adipocytes	*Gymnema inodorum* extract + Phosphatidylcholine (PC)	NR	PS- 164 to 180 PDI- <0.5 EE- 41.06 ± 6.26 ZP- −35 to −45	*Gymnema inodorum* extract nanophytosomes improved solubility and anti-inflammatory activity while retaining anti-insulin resistance, indicating potential for pharmaceutical applications.	[68]
18	*Cicer arietinum*-enriched polyunsaturated fatty acids	Hepatosteatosis	*Cicer arietinum* + phosphatidylcholine (soy lecithin)	NR	EE- 99.57	Phytosomes enhanced therapeutic efficacy over the pure drug by improving liver function, lipid profile, and oxidative stress, indicating their potential as an effective delivery system.	[69]
19	Eucalyptus Oil	To check the Effect of Cholesterol and Different Solvents on Particle Size and Drug Release	Eucalyptus Oil + Soya Lecithin + Cholesterol	NR	PS- 71.76 ± 0.63 to 197.36 ± 0.53 nm EE- 57.45 ± 0.35 to 67.34 ± 0.52 ZP- 15.3 ± 0.27 to −28.2 ± 0.26	Preparation of Eucalyptus oil phytosomes demonstrated that the solvent and cholesterol had a major impact on particle size and stability, underscoring key formulation factors for optimal delivery.	[70]
20	*Spirulina platensis*	Wound Healing	*Spirulina platensis* + L-α phosphatidylcholine + cholesterol	Topical	PS- 598.40 ± 9.68 PDI- 0.62 ± 0.07 EE- 62.76 ± 1.75 ZP- −19.8 ± 0.49	S. platensis nanophytosome gel has shown improved wound treatment by enhancing wound healing, anti-inflammatory, antioxidant, and angiogenic activities with superior effectiveness.	[47]
Thin-film hydration method + Probe sonication							
21	Carvacrol	To enhance the cryotolerance of Buffalo semen	Carvacrol + Phospholipon 90H	NR	PS- 286.7 ± 11.27 PDI- 0.189 ± 0.05 ZP- −11.4 ± 0.26	Carvacrol-loaded Phytosome improved Sperm quality, integrity, oxidative stress, and conception rates, suggesting a promising future for semen cryopreservation and reproductive uses.	[71]
22	*Mesua ferrea* Linn. Extract	Atopic dermatitis	*Mesua ferrea* Linn. Extract + soya lecithin + cholesterol	Topical	PS- 72.16 ± 2.38 PDI- 0.22 ± 0.08 EE- 60.71 ± 0.47 ZP- 50.36 ± 0.43	*Mesua ferrea* Linn. Extract-loaded phytosome gel increased skin permeability and anti-atopic dermatitis activity.	[31]
23	Diosmetin	Comparison of solubility and in vitro drug release of Diosmetin to its Phytosomal formulation	Diosmetin + cholesterol + soy lecithin	NR	PS- 213.9 PDI- 0.293 EE- 95.60 ZP- −115.1	Diosmetin-Phytosomes demonstrated significant potential as an effective oral carrier system, improving stability, bioavailability, and safety.	[56]
24	Vitamins A, E, D, B complex, folic acid, C, and zinc	Liver and kidney injury	Multivitamins + soy lecithin	Oral	PS- 149.4 ± 1.258 PDI- 0.344 ±0.064 ZP- −34.3 ± 1.253	Nanophytosomes loaded with vitamins demonstrated durability and compatibility without compromising product quality, suggesting their potential immune-boosting properties.	[72]
25	Aristatoside C (AC) and Davisianoside B (DB)	Hemolytic Activity	Extract + soy lecithin + cholesterol	NR	With AC PS- 121.06 ± 1.01 PDI- 0.248 ± 0.004 EE- 68.0 ± 5.2 ZP- −106.8 ± 0.4 With DB PS- 110.64 ± 0.37 PDI- 0.255± 0.014 EE- 79.5 ± 3.1 ZP- −88.1 ± 2.8	Phytosomes retained cytotoxicity, exhibited reduced hemolysis, and enabled sustained release, indicating improved safety and potential as anticancer nanotherapeutics.	[73]
26	Resveratrol + Quercetin	For scar management	Resveratrol + quercetin + phosphatidylcholine	NR	PS- 110 EE- 70 ZP- −29	In a siloxane gel, resveratrol-quercetin phytosomes improved drug loading, release, and skin absorption, thereby increasing hydration and therapeutic effectiveness in scar therapy.	[74]
27	*Curcuma longa L*. (CL) + *Plumbago zeylanica L* (PZ) extracts	Wound healing	Extract + Soy phosphatidylcholine	Topical	PS- 241 PDI- 0.239 ZP- −27	Phytosomal gel demonstrated superior wound-healing efficacy through increased stability, enhanced penetration, and prolonged release. In comparison to traditional formulations	[75]
28	Carvacrol	Wound healing	Carvacrol + Lipoid S100 and Phospholipon 90H	Topical	PS- 120.5 ± 1.99 PDI- 0.206 ± 0.045 EE- 96.57 ± 3.36 ZP- −11.7 ± 1.969	The carvacrol-loaded phytosomal formulation enhanced release, penetration, and wound healing, suggesting it could be an effective topical treatment.	[76]
29	Silymarin	Hepatoprotective effect	Silymarin + soybean phosphatidylcholine	Oral	PS- 105.16 ± 7.303 PDI- 0.374 ± 0.07 EE- 96 ZP- −10.681 ± 1.6	Silymarin phytosome nanoparticles improved hepatoprotective benefits by reducing oxidative stress, inflammation, and lipid peroxidation compared with the bulk medication.	[77]
30	Bergamot Essential Oil	Acne Vulgaris	Bergamot Essential Oil + phosphatidylcholine (soya) + cholesterol	Topical	PS- 300.40 ± 22.56 PDI- 0.571 ± 0.16 EE- 87.89 ± 4.14 ZP- −29.7 ± 1.54	Bergamot Essential Oil-loaded Phytosome had improved characteristics and greater anti-acne activity,	[78]
31	Phenolic-enriched grape seed Extract	To improve stability, gut absorption, and oral bioavailability	Extract + Soybean lecithin + Cholesterol	Oral	PS- 168.6 ZP- 58.86 ±1.22	Cholesterol-incorporated, polysaccharide-coated nanophytosomes enhanced the stability, controlled release, and bioavailability of Vitis vinifera phenolics.	[79]
32	Andrographolide	To enhance cellular delivery and pro-apoptotic activities in HepG2	Andrographolide + L-α-phosphatidylcholine	NR	PS- 243.7 ± 7.3 PDI- 0.31 EE- 72.20 ± 4.53	Andrographolide-loaded phytosomes enhanced anticancer activity against HepG2 cells by inducing G2/M arrest and mitochondrial apoptosis, thereby improving therapeutic efficacy.	[80]
Thin-film hydration method + Ultrasonication							
33	Pomegranate Fruit Extract	Anti-oxidant	Pomegranate Fruit Extract + soya Phosphatidylcholine + Cacl	NR	PS- 128.6 PDI- 0.168 EE- 98.53 ZP- −40.15	Pomegranate nanophytosomes demonstrated superior functional performance, including increased stability, enhanced antioxidant activity, and controlled release.	[54]
34	Rubiacordifolia Extract	Neuropathic Pain	Rubiacordifolia Extract + L-α-phosphatidylcholine	Oral	PS- 289.1 ±0.21 EE- 72.12 ZP- −6.92 ± 0.10	Rubiacordifolia extract phytosomes enhanced bioavailability, drug release, and neuroprotective efficacy, indicating potential for effective management of neuropathic pain.	[55]
35	Parthenolide	Gentamicin-Induced Nephrotoxicity	Parthenolide + phosphatidylcholine (soya)	Oral	PS- 407.4 PDI- 0.6	Parthenolide-Phytosomes showed dose-dependent nephroprotection via activation of the Sirt1/Nrf2/HO-1/NQO1 pathway, with good safety, indicating potential as a protective adjunct in gentamicin therapy.	[81]
Thin-film hydration method + Bath sonication							
36	Extracts of ginger (GINex) and Rosehips (ROSAex)	Antioxidant and Anti-inflammatory	Extracts + soy phosphatidylcholine (PC)	Oral	PS- 119.95 ± 8.64 to 783.14 ± 219.26 PDI- 0.39 ± 0.02 to 0.56 ± 0.06 EE- 83.3 and 94.4 ZP- −18.17 ± 1.85 to −22.66 ± 2.44	Ginger-rosehip phytosomes enhanced bioavailability and synergistically enhanced antioxidant and anti-inflammatory effects, with improved safety.	[82]
Thin-film hydration method + High-speed homogenization							
37	Hinokitiol	Breast Cancer	Hinokitiol + Phospholipon 90 G + Cholesterol + sodium deoxycholate	NR	PS- 138.4 ± 7.7 to 763.7 ± 15.4 PDI- 0.449 ± 0.02 to 0.848 ± 0.045 EE- 29.161 ± 1.163 and 92.77 ± 7.01 ZP- −10.2 ± 0.28 to −53.2 ± 1.06	Hinokitiol phytosomes increased cytotoxicity, regulated release, and induced apoptosis, suggesting greater targeting potential and anticancer effectiveness.	[30]
Thin film hydration method + Homogenization + Probe sonication							
38	Rutin	Anti-Tumour, Antioxidant, and Anti-Inflammatory Activity in Ehrlich Ascites Carcinoma	Rutin + Phosphatidyl choline + Cholesterol	NR	PS- 185 PDI- 0.392 EE- 84.62 ZP- −37.0	Rutin-phytosome nanoparticles demonstrated greater anticancer activity than crude, improving tumour inhibition, normalising biomarkers, and enhancing antioxidant status.	[32]
Anti-solvent precipitation technique							
39	*Pongamia pinnata* leaves	Antidiabetic	*Pongamia pinnata* + Phospholipid +Cholesterol	NR	PS- 138.95 EE- 73.12 ZP- −45.65	Pongamia pinnata phytosomes enhanced bioavailability and antidiabetic activity, demonstrating efficacy comparable to that of glibenclamide.	[35]
40	*Tinospora cordifolia*	Anti-Inflammatory	*Tinospora cordifolia* + Cholesterol	NR	PS- 76 μm EE- 72.34–95.91	Phytosomes demonstrated an effective delivery strategy by improving bioavailability and permeability and enabling controlled release.	[53]
41	*Acalypha indica*, *Pergularia daemia* and *Coleus amboinicus*	Pharyngitis	Polyherbal + soy lecithin	Topical	PS- 158 EE- 86.94 ± 0.3 ZP- −8	Phytosomes enhanced bioavailability and delivery, and demonstrated anti-inflammatory and antimicrobial activity compared to the standard.	[33]
42	*Sonneratia alba* extract	Antimalarial activity	*Sonneratia alba* extract + soy lecithin	NR	PS- 471.8 EE- 82.40 ZP- −54.1	Phytosomes increased solubility, stability, and antimalarial activity compared to the extract and the fraction.	[83]
43	Adiantum capillus-veneris	Antimicrobial activity	*Adiantum capillus-veneris* + phosphatidylcholine	NR	PS- 187 PDI- 0.2421 EE- 97–98.4 ZP- −17.22	A phospholipid complex improved dissolution, bioavailability, and therapeutic efficacy.	[36]
Anti-solvent precipitation technique + Probe sonication							
44	Quercetin	To Improve Skin Delivery	Quercetin + Phospholipon^®^ 90 G	Topical	PS- 206.7 EE- 85.30 ZP- −26.3	Skin penetration was enhanced by optimised phytosomes, which exhibited acceptable stability and tolerability, suggesting potential for successful dermal delivery.	[84]
Co-solvency method							
45	Curcumin	To improve the solubility, in vitro digestive and Thermal Stability of the drug.	Curcumin + Lipoid P75 + Maltodextrin	NR	PS- 2.81 ± 0.19 to 26.42 ± 4.06 µm EE- 86.50 ± 0.25 to 93.97 ± 0.27	Spray-dried curcumin phytosomes improved solubility and in vitro digestive and thermal stability, indicating enhanced formulation performance.	[38]
46	Mastoparan-M (Mast-M)	Breast cancer	Mast-M + Soya phosphatidylcholines	NR	PS- 125.67 ± 45.79 ZP- +32.05 ± 17.27	Phytosome-Mast-M demonstrated safe and efficient anticancer activity, suggesting great promise as a new nanomedicine for breast cancer treatment.	[57]
47	Verbascoside from the *Lantana camara* crop	Antioxidant and Hypoglycemic	verbascoside + Phosphatidyl choline from soy lecithin + cholesterol	NR	PS- 136.2 PDI- 0.13 EE- 65.3 ± 2.1	The stability, controlled release, and bioavailability of phenolics were enhanced by phytosomes, suggesting potential uses as hypoglycemic agents and antioxidants.	[40]
48	Dragon fruit extract (*Hylocereus costaricensis*)	Antioxidant, Antihyperglycemic, and Anti-inflammatory	*Hylocereus costaricensis* + Phosphatidylcholine (60% purified from the yolk of egg)	Oral	PS- 1329 ± 121.0 PDI- 0.633 ± 0.039 EE- 46 ZP- −16 ± 1	Dragon fruit phytosomes exhibited enhanced bioactivity at lower doses, with improved antihyperglycemic and anti-inflammatory effects, indicating greater bioavailability and efficacy.	[37]
49	Methyl jasmonate-treated *D. Morbifera* adventitious root extract (MeJA-DMARE)	Anti-Inflammatory	MeJA-DMARE + Soy-L-α-phosphatidylcholine	NR	PS- 210 PDI- 0.16 EE- 79.98 ± 1.45 ZP- −25.19	MeJA-DMARE phytosomes demonstrated greater anti-inflammatory and anticancer activity by increasing bioactivity, solubility, and permeability, with minimal toxicity.	[41]
50	α-Mangostin	Ovarian cancer	α-Mangostin + Phospholipon-90G	NR	PS- 138.9 PDI- 0.221 EE- 95.10 ZP- −44.5 ± 1.5	In SKOV-3 cells, α-M phytosomes increased cytotoxicity and mitochondrial-mediated apoptosis, suggesting greater anticancer efficiency.	[39]
Co-solvency method + Ultrasonication							
51	Green tea extract	Coccidiosis infection	Green tea extract + lecithin	Oral	PS- 344 ± 82.5 PDI- 0.86	Green tea phytosomes were superior to the extract in anticoccidial activity and gut health at lower doses, indicating improved therapeutic efficacy.	[85]
Mechanical dispersion/emulsification approach							
52	*Pentaclethra macrophylla* seed extract	Anti-inflammatory activity	*Pentaclethra macrophylla* + Phospholipon 90 H	Oral	EE- 92.99 ± 0.09	Phytosomes demonstrated improved in vivo anti-inflammatory activity, prolonged drug-release kinetics, and high entrapment efficiency, suggesting remarkable therapeutic potential.	[43]
53	*Yerba mate* (*Ilex paraguariensis*)	A systematic preparation	Yerba mate + soya phosphatidylcholines	NR	PS- 518.6 ± 16.0 PDI- 0.589 ZP- −47.5 ± 0.5	Yerba mate-loaded lipid vesicles showed reduced size and enhanced stability via phenolic-phospholipid interactions, indicating potential phytosome formation and improved delivery.	[86]
54	Geraniin	To Enhance the Bioavailability of Geraniin	Geraniin+ lecithin Lalpha-phosphatidylcholine (Lipoid EPC) or Soy phosphatidylcholine (SPC)	Oral	PS- 1.04 ± 0.11 µm PDI- 0.26 ± 0.02 EE- 100.2 ± 0.8 ZP- −26.1 ± 0.12	A phospholipid complex improved pharmacokinetics, increasing lipophilicity and oral bioavailability (11- and 5-fold increases in plasma levels), suggesting improved delivery efficacy.	[58]
55	Curcumin	Parkinson’s disease	Curcumin + phosphatidylcholine	NR	PS-197.15 ± 4.42 PDI- 0.121 ± 0.032 EE- 26.00 ZP- −18.2 ± 2.0	Curcumin-loaded nanophospholipid complexes improved neuroprotective effectiveness and mitochondrial pathway markers (Parkin, PINK1, and BAG5).	[42]
56	*Callistemon citrinus* Leaf Extract	Obesity	*Callistemon citrinus* + soybean phospholipids	Oral	PS-129.98 ± 18.30 EE- 80.49 ± 0.07	Phytosomes enhanced solubility, bioavailability, and antioxidant activity, resulting in a 40% reduction in body weight and improved therapeutic efficacy.	[44]
57	*Nicotiana tabacum* var. Virginia Leaf Extract	Antioxidant, anti-inflammatory, and attenuating intracellular reactive oxygen species activities	Extract + phosphatidylcholine + Cholesterol	NR	PS- 311.00 ± 5.21 to 163.40 ± 0.26 PDI- 0.234 ± 0.017 to 0.098 ± 0.015 ZP- −22.57 ± 0.23 to −76.93 ±1.71	Phytosomes increased antioxidant and anti-inflammatory activity by lowering intracellular ROS and nitric oxide levels, while maintaining high stability, suggesting promise for skin protection and anti-ageing applications.	[87]
58	*Tinospora cordifolia* Extract (Epiyangambin)	Anti-diabetic, anti-inflammatory, and analgesic activity	Extract + cholesterol and soya-lecithin	Oral	PS- 454.2 ± 8.1 PDI- 0.33 EE- 82.7 ± 1.6 ZP- −43.1 ± 7.5	Phytosomes showed superior therapeutic efficacy, including greater solubility, sustained release (Fickian diffusion), and anti-diabetic, anti-inflammatory, and analgesic actions, compared to the extract.	[34]
59	Amphotericin B	To develop an infant-friendly SNEDDS	Amphotericin B + monoacyl-phosphatidylcholine	Oral	PS- 179 ± 1 PDI- 0.22 ± 0.00 ZP- 71.4 ± 0.3	Amphotericin B-phospholipid complex-loaded SNEDDS demonstrated improved oral delivery for pediatric use by enhancing solubilization and dissolution, enabling rapid nanoemulsion formation.	[88]
60	*Allium ampeloprasum* Extract	Breast cancer	Extract + phospholipid	Oral	PS- 226 PDI- 0.33 ZP- −48.65	Nanophytosomes demonstrated enhanced anticancer efficacy by improving redox balance, inducing apoptosis (Bax, caspase-3, Bcl2), and inhibiting tumour development.	[89]
Mechanical dispersion/emulsification approach + Ultrasonication							
61	*Callistemon citrinus* Extract	Oxidative stress in the brain	*Callistemon citrinus* Extract + soybean phosphatidylcholine	Oral	PS- 129.98 ± 18 EE- 80.49 ± 0.07	Phytosomes restored antioxidant enzyme activity (SOD, CAT, GSH) and reduced brain oxidative stress at lower doses than the extract, indicating greater neuroprotective efficacy.	[90]
Mechanical dispersion/emulsification approach + Ultrasonication + High-pressure homogenization							
62	Casein	Effects of homogeneous and ultrasonic treatment on casein/phosphatidylcholine complex-emulsions	Casein+ phosphatidylcholine	NR	PS- 130.20 ± 3.96 to 792.93 ± 37.76 ZP- −24.50 ± 0.87 to −40.13 ± 1.12	Ultrasonically treated casein complex showed enhanced solubility, stability, bioactivity, emulsification, and digestion compared with homogeneous treatment.	[91]
Microfluidic and continuous flow systems							
63	Curcumin	To improve the oral bioavailability of curcumin–phospholipid complex	Curcumin + soy lecithin	Oral	PS- 71.19 ± 1.37 PDI- 0.226 ± 0.047 ZP- −38.23 ± 0.89	Curcumin Complex outperformed curcumin in oral bioavailability and pharmacokinetics (Cmax: 2163 vs. 133 ng/mL; AUC: 9494 vs. 937 ng/mL/h), indicating greater systemic exposure.	[92]
64	*Stellaria media*	To improve skin delivery	Stellaria media + Lipoid S 100	Topical	PS- 89 ± 1 PDI- <0.25 EE- 72 ± 1.8 ZP- −38.0 ± 1.5	Phytosome enhanced skin permeation (2-fold) with controlled release and stability, compared to the pure form, indicating improved dermal delivery.	[51]
Melting–Hydration							
65	Triterpine	Acute kidney injury	Tripterine + Lipoid S100 + Selenium	Oral	PS- 200 ZP- −38.5	Selenized tripterine phytosomes enhanced antioxidant capacity and reduced cytotoxicity while inhibiting ferroptosis via GPX4 and DUSP1 pathways.	[48]
Membrane dispersion–sonication method							
66	Neohesperidin	To amplify neohesperidin bioavailability	Neohesperidin + cholesterol+ soybean phosphatidylcholine+ HP-β-CD	NR	PS- 96.7 to 132.2 PDI- <0.2 EE- 92.3 ± 1.2 to 95.6 ± 1.2 ZP- −9.5 ± 0.28 to −11.3 ± 0.25	Phospholipid-cyclodextrin nanosystems enhanced solubility (2-fold), bioaccessibility (66% vs. 21%), and antioxidant activity compared to free Neohesperidin, indicating improved bioavailability and stability.	[49]
Ethanol injection method + Rotary evaporator + Ultrasonication							
67	Fisetin	Breast cancer	Fisetin + Soy phosphatidylcholine (Lipoid S100) + Cholesterol +stearylamine (SA) tocopheryl polyethylene glycol 1000 succinate (TPGS) + Hyaluronic acid (HA)	Intravenous	PS- 226.67 ± 2.01 to 277.76 ± 25.58 PDI- 0.19 ± 0.02 to 0.44 ± 0.06 ZP- −21.73 to +51.43	Modified fisetin cholephytosomes enhanced solubility and sustained release, with 10-fold greater cytotoxicity than the free drug, indicating superior anticancer efficacy.	[50]

PS = particle size; PDI = polydispersity index; EE = entrapment efficiency; ZP = zeta potential. particle size is reported in nanometres (nm) unless otherwise specified. SPC, soybean phosphatidylcholine; EPC, egg phosphatidylcholine; PC, phosphatidylcholine; PBS, phosphate-buffered saline; snedds, self-nanoemulsifying drug delivery system; MVLS, multivesicular liposomes; Cmax, maximum plasma concentration; AUC, area under the plasma concentration-time curve. Data are presented as reported in the original studies. where a range of values is provided, it represents the variation observed among different formulations or experimental conditions within the same study. NR indicates that the authors did not report the information. The PS, PDI, EE, and ZP columns represent the characterisation parameters considered in this review; however, individual studies did not necessarily report all four parameters. Accordingly, only the values reported by the original authors are presented for each study. Abbreviations used in phytosome composition and characterisation are defined according to the respective original references.

Higuchi kinetics have been reported for neohesperidin phytosomes [49], Korsmeyer-Peppas kinetics for hyaluronic acid-coated PEGylated fisetin cholephytosomes [50], Weibull kinetics for phytosomal formulations reported by [93] and first-order kinetics [19]. Similarly, Pongamia pinnata phytosomes showed good agreement with both the Higuchi and Korsmeyer-Peppas models, indicating diffusion-controlled release [35].

Whereas verbascoside phytosomes followed Higuchi, Weibull, and Korsmeyer-Peppas kinetics, suggesting multiple release pathways arising from complex drug-phospholipid interactions [40]. Overall, the in vitro release behaviour of phytosomal systems is governed by the combined influence of formulation composition, particle characteristics, drug loading, entrapment efficiency, and drug-phospholipid interactions rather than by any single formulation parameter. This flexibility enables the rational design of phytosomal formulations with immediate, controlled, or sustained-release profiles to meet specific therapeutic requirements. Consequently, optimised release characteristics not only improve solubility, absorption, and bioavailability but also protect labile bioactives from premature degradation, thereby enhancing therapeutic performance and supporting the broader application of phytosomes across diverse disease conditions.

## 6. Bioavailability Enhancement and Pharmacokinetic-Pharmacodynamic Relationship

The physicochemical characteristics discussed in the previous sections ultimately determine the in vivo performance of phytosomal systems. Phospholipid composition, bioactive phospholipid complexation, particle size, surface characteristics, and formulation stability can influence dispersion, membrane interactions, release behaviour, and subsequent absorption. These formulation-dependent properties can, in turn, affect pharmacokinetic parameters, such as Cmax, Tmax, AUC, and tissue distribution, by increasing membrane affinity, protecting bioactives from degradation, and modulating release behaviour, thereby potentially contributing to a more favourable therapeutic effect [94]. The enhanced absorption and systemic exposure resulting from this phyto-phospholipid complexation are shown in Figure 5, with phytosome increasing membrane interactions, endocytic uptake, and partial lymphatic transport, and decreasing first-pass metabolism. These proposed mechanisms provide a basis for understanding how formulation characteristics may influence systemic exposure, although their contribution can vary among phytosomal systems. The results of the reported studies are consistent with a marked increase in systemic uptake following phytosomal complexation.

For instance, geraniin-phospholipid complexes showed about 5-fold and 11-fold increases in plasma Cmax at the first and second absorption peaks, respectively, and 8-fold and 10-fold increases in AUC0-t and AUC0-∞, respectively, suggesting significantly improved absorption and longer systemic retention [58]. Similarly, the phytosome formulation of Rubia cordifolia for neuropathic pain led to a 2.25-fold increase in Cmax and a 134.14% increase in relative bioavailability compared with the crude extract, demonstrating improved intestinal absorption and systemic bioavailability [55]. The magnitude of these improvements varies among bioactives and formulation strategies, indicating that enhanced exposure is not a uniform feature across all phytosomal systems.

There is often a correlation between an improved pharmacokinetic performance and a better tissue distribution. For example, plasma levels of 6-gingerol were elevated 2.6-fold in the liver and 40-fold in the kidney in rats fed ginger rhizome-rosehip phytosomes compared with the non-complexed extract, resulting in greater antioxidant and anti-inflammatory effects [82]. Thus, indicating that, in addition to improved absorption, phytosomal complexation may also enhance the delivery of bioactives to target tissues.

The effect of formulation properties on bioavailability enhancement is particularly noticeable in nanoscale phytosomal systems. For example, microfluidic-prepared curcumin–phospholipid complex nanoparticles resulted in a Cmax of 2163.87 ng/mL and an AUC0-t of 9494.28 ng/h/mL, compared with 133.60 ng/mL and 936.99 ng/h/mL, respectively, for free curcumin [92]. These values correspond to approximately 16.2-fold and 10.1-fold increases in Cmax and AUC0-t, respectively. These enhancements were attributed to reduced particle size, improved dissolution characteristics, enhanced membrane permeability, and increased intestinal absorption. The combination of phospholipid complexation with microfluidic nanoparticle engineering therefore illustrates how formulation strategy can influence systemic exposure, pharmacokinetic behaviour and therapeutic performance (Figure 6A–C).

Furthermore, aprepitant-phospholipid complex-loaded intravenous emulsions, which exhibited about a 3-fold higher AUC and better liver targeting than conventional formulations, demonstrated that phospholipid complexation can improve pharmacokinetic properties beyond those of oral delivery systems [63]. These pharmacokinetic improvements may contribute to pharmacodynamic benefits; however, the extent of this relationship depends on the bioactive, formulation, systemic exposure, and response relationships, as well as the clinical context. In patients with osteoarthritis, curcumin phytosomes were more effective at reducing inflammation and pain [95]. The hepatoprotective activity of the silymarin phytosomes was demonstrated by modulating liver enzymes and oxidative stress biomarkers [96]. Similarly, Ginkgo biloba phytosomes showed greater plasma exposure and antioxidant and neuroprotective activity than the extract [97]. These examples demonstrate that phytosomes not only help raise the drug’s blood levels but also optimise the therapeutic profile of bioactives by influencing their absorption, distribution, metabolism, and elimination. These changes may enhance pharmacological effects, although improved exposure does not necessarily translate into proportional or clinically meaningful therapeutic benefit. This PK-PD relationship provides a useful framework for linking formulation design and physicochemical properties with biological outcomes, while clinical relevance requires confirmation through appropriately designed human studies.

## 7. Therapeutic Applications of Phytosomal Drug Delivery Systems

The therapeutic relevance of phytosomal systems arises from their ability to modify the delivery of poorly bioavailable natural bioactives. Improvements in solubility, membrane interaction, systemic exposure, and tissue distribution can influence the pharmacokinetic and biological behaviour of these compounds. Consequently, phytosomal formulations have been investigated for their effects on metabolic regulation, inflammation, oxidative stress, immune responses, cellular protection, and tissue repair across a range of conditions, including metabolic and neurological disorders, cancer, infectious diseases, organ protection, autoimmune disorders, and wound healing. The extent of these effects varies with the bioactive, formulation, and therapeutic context.

Emerging strategies, including targeted delivery, controlled release, hybrid systems, AI-assisted formulation design, and personalised approaches, are further being explored to tailor phytosomal delivery to specific therapeutic requirements (Figure 7).

### 7.1. Metabolic Disease Management

#### 7.1.1. Antidiabetic Applications

Phytosomal delivery systems have demonstrated significant potential in improving glycaemic control and metabolic regulation. Phytosome formulations of Tinospora cordifolia, C. aronia, and other medicinal plant extracts have shown enhanced glucose-lowering efficacy in streptozotocin-induced diabetic models, often producing therapeutic outcomes comparable to standard antidiabetic drugs such as metformin and glibenclamide [34,35,98]. These improvements are largely attributed to enhanced intestinal absorption, increased systemic exposure, and improved tissue distribution of the encapsulated bioactive.

Similar benefits have been reported for several phytochemicals with established metabolic activity. Curcumin phytosome formulations have demonstrated significant improvements in glycaemic control, insulin sensitivity, and metabolic parameters in subjects with impaired fasting glucose and prediabetes [99]. Likewise, quercetin phytosome formulations have shown substantially enhanced oral absorption and systemic exposure in healthy volunteers compared with conventional quercetin preparations, overcoming one of the major limitations associated with its therapeutic application [100]. The improved pharmacokinetic performance achieved through phytosomal delivery may enhance metabolic efficacy by increasing the bioavailability of phytoconstituents at target tissues.

Mechanistically, phytosomal formulations may exert antidiabetic effects through multiple complementary pathways, including enhancement of insulin signalling, improvement of glucose uptake, attenuation of oxidative stress, suppression of chronic inflammatory responses, preservation of pancreatic β-cell function, and modulation of cellular energy metabolism. Enhanced bioavailability facilitates greater interaction of phytoconstituents with key metabolic regulators, thereby improving glycaemic control and reducing diabetes-associated metabolic dysfunction. Collectively, the available evidence supports the use of phytosomal technology as a promising strategy to enhance the therapeutic efficacy and translational potential of antidiabetic phytoconstituents.

#### 7.1.2. Anti-Obesity and Metabolic Regulation

The enhanced bioavailability achieved through phytosomal encapsulation has also translated into improved anti-obesity activity. Multiple studies have reported reductions in body weight gain, adiposity indices, dyslipidemia, and oxidative stress markers following administration of phytosomal formulations of quercetin and Callistemon citrinus extracts [44,59]. These findings suggest that phytosomes represent promising delivery systems for managing obesity and associated metabolic disorders. Similar benefits have been observed with other phytoconstituents possessing metabolic regulatory properties. Bergamot polyphenolic phytosome formulations have shown favourable effects on serum lipid parameters, metabolic syndrome-associated biomarkers, and cardiovascular risk factors, supporting their potential role in obesity-associated metabolic disorders [101]. In addition, grape seed phytosomal preparations have been reported to improve lipid metabolism, reduce oxidative stress, and attenuate obesity-related inflammatory responses through enhanced systemic bioavailability [102]. Collectively, these findings suggest that phytosomal delivery systems may be an effective strategy to improve the metabolic efficacy of phytoconstituents and facilitate their application in obesity management.

### 7.2. Immunomodulatory and Anti-Inflammatory Therapy

#### 7.2.1. Systemic Anti-Inflammatory Applications

Inflammation is a central driver of numerous chronic diseases, and phytosomal systems have been widely investigated for enhancing the anti-inflammatory activity of plant-derived bioactives. Enhanced absorption, prolonged circulation, and improved tissue distribution have led to superior suppression of inflammatory mediators, oxidative stress, and tissue injury compared with the corresponding free phytoconstituents [43,61]. Several anti-inflammatory phytoconstituents have shown substantial benefits from phytosomal delivery. Boswellic acid phytosomal formulations have demonstrated enhanced anti-inflammatory efficacy by inhibiting 5-lipoxygenase-mediated leukotriene synthesis and suppressing chronic inflammatory responses [26]. Similarly, apigenin phytosomal systems have shown improved inhibition of NF-κB signalling and reduced production of pro-inflammatory cytokines, including TNF-α, IL-1β, and IL-6 [52]. Naringenin [103] and epigallocatechin gallate (EGCG)-based [24] phytosomal formulations have further demonstrated superior antioxidant and anti-inflammatory activities, driven by enhanced bioavailability and tissue exposure.

Mechanistically, phytosomal formulations enhance anti-inflammatory efficacy by facilitating greater interaction of phytoconstituents with key inflammatory pathways. Reported mechanisms include suppression of NF-κB activation, inhibition of cyclooxygenase and lipoxygenase pathways, attenuation of reactive oxygen species generation, modulation of cytokine networks, and restoration of cellular redox homeostasis. Collectively, these findings support the use of phytosomal technology as an effective strategy for improving the therapeutic efficacy of anti-inflammatory phytoconstituents.

#### 7.2.2. Autoimmune and Immune-Mediated Disorders

Beyond general anti-inflammatory effects, phytosomal formulations have demonstrated immunomodulatory activity in autoimmune and chronic inflammatory diseases. Studies have shown regulation of cytokine production, suppression of pathogenic immune responses, and restoration of immune homeostasis in experimental models of arthritis [26], colitis [31], dermatitis and psoriasis [20]. Boswellia phytosomal formulations have shown beneficial effects in osteoarthritis and rheumatoid arthritis by suppressing inflammatory signalling pathways and reducing joint inflammation [26]. Likewise, baicalin phytosomes have demonstrated immunoregulatory activity in inflammatory bowel disease models by modulating cytokine production and attenuating mucosal inflammation [48]. Hesperidin and oleuropein-based phytosomal systems have also demonstrated protective effects against chronic inflammatory disorders by regulating oxidative stress, macrophage activation, and immune signalling pathways [104]. Mechanistically, phytosomal formulations may contribute to immune homeostasis by regulating innate and adaptive immune responses, modulating macrophage polarisation, suppressing pro-inflammatory cytokine networks, enhancing antioxidant defence systems, and restoring tissue immune balance. These findings highlight the potential of phytosomes as delivery platforms for immune-targeted phytotherapeutics and chronic inflammatory disease management.

### 7.3. Anticancer Drug Delivery

Cancer therapy remains one of the most extensively explored applications of phytosomal technology. Numerous phytosome formulations have demonstrated enhanced tumour suppression, improved intracellular uptake, prolonged circulation, and greater therapeutic efficacy than their free phytoconstituent counterparts. Significant reductions in tumour growth, tumour burden, and metastatic progression have been reported across multiple experimental cancer models, including breast cancer [57] and colorectal cancer [28]. Recent studies have further demonstrated that phytosomal delivery can improve systemic retention, tumour accumulation, and therapeutic efficacy in vivo. For example, phytosomal Mastoparan-M exhibited prolonged circulation, enhanced tumour localisation, significant inhibition of tumour growth, and increased apoptotic cell death in a 4T1 breast cancer model, highlighting the ability of phytosomal systems to improve both pharmacokinetic and therapeutic performance.

Several phytoconstituents with established anticancer activity have benefited substantially from phytosomal delivery. Berberine phytosomal formulations have demonstrated enhanced antiproliferative and pro-apoptotic activity in breast and colorectal cancer models [105]. In contrast, genistein phytosomal systems have shown improved bioavailability and tumour-suppressive efficacy in Breast cancers [106]. Resveratrol phytosomes have also demonstrated enhanced inhibition of tumour growth, angiogenesis, and metastatic progression by improving intracellular accumulation and prolonging systemic exposure [107]. Similarly, fisetin-containing cholephytosomes demonstrated significant antitumor efficacy against breast cancer by modulating TGF-β1, ERK1/2, NF-κB, and MMP-9 signalling pathways [50]. Overall, phytosomal delivery enhances anticancer efficacy by improving tumour accumulation, increasing intracellular uptake, inducing apoptosis, suppressing proliferative signalling pathways, inhibiting angiogenesis, modulating oxidative stress responses, and reducing metastatic potential. Enhanced delivery of phytoconstituents facilitates greater interaction with molecular targets involved in tumour progression, thereby improving therapeutic outcomes while potentially reducing systemic toxicity. Collectively, these findings indicate that phytosomal systems can effectively overcome many pharmacokinetic limitations associated with anticancer phytochemicals and may contribute to the development of safer and more effective plant-based anticancer therapeutics.

### 7.4. Anti-Infective Delivery Applications

#### 7.4.1. Antibacterial Therapy

Phytosomal formulations have been shown to improve antimicrobial efficacy by enhancing solubility, membrane interactions, and local tissue penetration. Enhanced antibacterial activity has been reported against a broad spectrum of clinically relevant pathogens, demonstrating the ability of phytosomes to improve therapeutic performance in infectious diseases [33,36]. Improved delivery facilitates greater interaction of phytoconstituents with bacterial membranes, resulting in enhanced disruption of microbial integrity and inhibition of bacterial proliferation. In several studies, phytosomal formulations exhibited superior antibacterial efficacy compared with corresponding free phytoconstituents, suggesting that improved bioavailability and tissue exposure contribute significantly to therapeutic outcomes.

#### 7.4.2. Antifungal and Antiparasitic Applications

Improved membrane affinity and controlled release characteristics have also facilitated the use of phytosomal systems in antifungal and antiparasitic therapy. Enhanced efficacy and reduced toxicity have been reported for several phytosomal formulations, including amphotericin B-based systems and phytoconstituent-loaded antiparasitic formulations [83,108]. Enhanced interaction with fungal and parasitic cell membranes may improve intracellular accumulation and therapeutic activity while minimising off-target toxicity. These findings highlight the potential of phytosomes as alternative or complementary anti-infective delivery platforms that can improve treatment efficacy and safety profiles.

### 7.5. Organ-Protective Drug Delivery Systems

#### 7.5.1. Hepatic and Pulmonary Protection

The ability of phytosomes to improve tissue exposure has facilitated their application in protecting major organs from oxidative stress and inflammatory injury. In liver disease models, phytosomal formulations have demonstrated significant hepatoprotective activity by restoring liver function markers, suppressing lipid peroxidation, and improving histopathological outcomes [69,77]. Enhanced antioxidant activity and improved bioavailability contribute substantially to these protective effects. Similarly, pulmonary protection has been achieved by attenuating inflammatory responses, reducing oxidative damage, and preserving lung architecture in experimental models of lung injury. For example, phytosome-encapsulated purple waxy corn tassel extract (PPT) was evaluated for its protective effects against PM2.5-induced pulmonary inflammation in male Wistar rats. PPT significantly suppressed the expression of cyclooxygenase-2 (COX-2), pro-inflammatory cytokines (IL-1β, IL-6, and IL-8), tumour necrosis factor-α (TNF-α), and nuclear factor kappa B (NF-κB) [109]. Likewise, pulmonary protection has been achieved by attenuating inflammatory responses and oxidative damage in lung injury models. Collectively, these findings support the utility of phytosomal systems for mitigating organ damage associated with chronic inflammation and oxidative stress.

#### 7.5.2. Cardiovascular and Renal Protection

Recent evidence also supports the use of phytosomal systems for cardiovascular and renal protection. Enhanced delivery of bioactive compounds has resulted in modulation of oxidative stress pathways, reduction in inflammatory signalling, preservation of organ function, and improvement of pathological outcomes in various cardiovascular and kidney disease models [48,110]. Reported benefits include improved endothelial function, attenuation of lipid peroxidation, reduction in tissue fibrosis, and preservation of renal architecture. These observations indicate that phytosomal delivery may enhance the therapeutic efficacy of phytoconstituents for the treatment of cardio-renal dysfunction and chronic degenerative diseases.

### 7.6. Neuroprotective and Brain Delivery Applications

#### 7.6.1. Neuroinflammatory Modulation

Effective treatment of neurological disorders often requires sufficient penetration across the blood–brain barrier. Phytosomal systems have shown promising capabilities to enhance central nervous system exposure and regulate neuroinflammatory pathways. Several studies have demonstrated reductions in oxidative stress, inflammatory cytokine production, and microglial activation following phytosomal treatment [90,111]. Improved bioavailability and tissue penetration facilitate enhanced interactions between phytoconstituents and neuroinflammatory signalling pathways, thereby attenuating neuronal damage and restoring neural homeostasis.

#### 7.6.2. Neurodegenerative Disorders

In neurodegeneration models, phytosomal formulations have improved neuronal survival, mitochondrial function, and cellular homeostasis. Enhanced neuroprotective effects have been reported in conditions associated with Parkinson’s disease, retinal degeneration, and age-related neurodegenerative processes [42,111]. Mechanistically, these effects have been attributed to the suppression of oxidative stress, the preservation of mitochondrial integrity, the modulation of apoptotic pathways, and the reduction in chronic neuroinflammation. Such findings highlight the potential of phytosomal delivery systems for addressing multiple pathological processes underlying neurodegenerative disease progression.

#### 7.6.3. Neuropathic Pain and Neural Repair

Beyond neuroprotection, phytosomal systems have demonstrated benefits for neuropathic pain and neural regeneration. Improved antioxidant defence, reduced neuronal damage, and enhanced functional recovery have been observed in experimental models of peripheral nerve injury and neuropathy [55]. Enhanced tissue penetration and sustained delivery may further contribute to improved neuronal repair, axonal regeneration, and restoration of neurological function following injury.

### 7.7. Tissue Regeneration and Wound Healing

Phytosomal systems have shown considerable potential in regenerative medicine by improving dermal penetration, local bioavailability, and sustained release of phytoconstituents. Enhanced wound closure, accelerated re-epithelialization, increased collagen deposition, improved angiogenesis, and modulation of inflammatory pathways have been consistently reported across wound-healing studies [47,75,76]. Improved tissue exposure facilitates greater interaction of phytoconstituents with cellular pathways involved in tissue repair, extracellular matrix remodelling, and regenerative responses. These findings support the application of phytosomal delivery systems in wound healing, skin regeneration, and broader regenerative medicine applications.

### 7.8. Emerging Precision Delivery Applications

Recent developments indicate that phytosomes are evolving from conventional bioavailability enhancers into advanced delivery platforms capable of precision therapeutic intervention. Emerging approaches include targeted phytosomal systems, controlled-release formulations, hybrid nanocarrier platforms, and multifunctional delivery systems designed to improve tissue specificity and therapeutic efficiency. These advances are expected to play an increasingly important role in the future development of personalised and precision phytomedicine [19,65]. The integration of stimuli-responsive systems, surface-functionalized phytosomes, and AI-assisted formulation design is expected to further enhance delivery precision and therapeutic performance.

## 8. Clinical Translation, Efficacy, and Safety of Phytosomal Formulations

In recent years, the number of clinical studies on phytosomal formulations has been steadily increasing (Table 2); however, phytosome research remains largely preclinical. The available clinical studies have investigated phytosomal formulations across metabolic, inflammatory, cardiovascular, gastrointestinal, sleep, and quality-of-life-related conditions. Most clinically evaluated formulations have been administered orally, either as dietary supplements or adjunctive treatments. The reported clinical evidence varies according to study design and intervention characteristics. Randomised controlled studies have reported improvements in clinical, physiological, and patient-reported outcomes with berberine phytosome, Melissa officinalis phospholipid-based extract, quercetin phytosome, cranberry phytosome, and coenzyme Q10 phytosome [112,113,114,115,116,117,118,119]. Observational studies have also reported changes in metabolic and biochemical outcomes, but provide comparatively weaker evidence for establishing treatment efficacy [116,120]. In addition, the interpretation of multicomponent interventions, such as quercetin phytosome combined with zinc and vitamin C, is limited because the individual contribution of the phytosomal component cannot be clearly distinguished from that of the other constituents [121]. These differences, together with variations in sample size, treatment duration, formulation composition, and outcome measures, limit direct comparison and generalisation across studies. Although improved absorption and systemic exposure have been reported for phytosomal formulations, particularly in preclinical and pharmacokinetic studies, these findings should not be equated with demonstrated clinical benefit, as increased systemic exposure does not necessarily translate into clinically meaningful improvements in patients. A further consideration for clinical translation is the predominance of phytosomal products as dietary supplements or adjunctive interventions rather than prescription medicines. Dietary supplements and botanical drugs follow different regulatory pathways, and the development of a botanical product as a therapeutic drug requires appropriate evidence of product quality, safety, and efficacy [122,123].

In addition, variability in botanical raw materials arising from differences in source, processing, and storage can affect phytochemical composition, formulation reproducibility, and performance, highlighting the importance of raw-material standardisation and quality control. Thus, while the reported studies indicate encouraging clinical potential and generally favourable short-term tolerability, the available evidence remains limited and heterogeneous and does not yet establish consistent clinical efficacy or long-term safety across phytosomal formulations. Further well-controlled, adequately powered, and longer-duration clinical studies are required to clarify therapeutic benefit, safety, and the broader clinical translation of phytosomal technology.

## 9. Emerging Innovations and Future Directions in Phytosome Technology

With the continued development of phytosome technology, research has shifted toward new functions beyond conventional bioavailability enhancement. Recent developments include improving targeting efficiency, developing alternative routes of administration, using computational tools to optimise formulations, and combining phytosomes with advanced nanocarriers. These innovations are part of the evolution of phytosomes from mere phospholipid complexes to multifunctional delivery systems capable of addressing more complex therapeutic needs.

### 9.1. Expanding Delivery Routes and Hybrid Nanocarrier Systems

Phytosomes, though predominantly formulated for oral delivery, have recently attracted attention for parenteral and pulmonary delivery to address problems with gastrointestinal absorption and first-pass metabolism. It has been possible to successfully formulate phytosome complexes into intravenous emulsions [19,91], soft nanoparticles [52], and inhalable delivery systems [103] to improve their bioavailability, tissue distribution, and targeted delivery to specific organs, including the brain, liver, and lungs. Simultaneously, incorporating phytosomes into other complementary nanocarriers to improve formulation performance is a focus of ongoing research [94]. A common strategy is to formulate phytosomes as nanosuspensions [92], nanoemulsions [91], or self-nanoemulsifying drug delivery systems (SNEDDS). These systems are hybrids of the membrane-compatibilizing effect of phytosomes and their high surface area and dissolution ability of nanoscale carriers. For instance, the nanosuspension formulation of silybin-phospholipid complex exhibited a better hepatoprotective effect and higher plasma levels than the standard phospholipid complex [124]. Likewise, the oral bioavailability of gentiopicroside phospholipid complexes was greatly enhanced by an SNEDDS-based delivery system [67]. These could be a powerful strategy to mitigate the biopharmaceutical challenge posed by poorly soluble bioactives.

### 9.2. Targeted and Controlled-Release Phytosome System

Surface engineering and hybrid carrier design for targeted and controlled-release systems are key concerns in current phytosome research. By functionalizing the surface with polymers, receptor-specific ligands, or bioactive coatings, selective accumulation of the phytosomal systems can be achieved at diseased tissues with minimal off-target exposure. For example, selenized tripterine phytosomes formulated to treat acute kidney injury exhibited greater renal accumulation and improved therapeutic efficacy via receptor-mediated uptake mechanisms [48]. Likewise, in breast cancer models, the modified phytosomes with hyaluronic acid showed greater receptor-mediated cell uptake, thereby improving treatment selectivity [106]. Apart from active targeting, there have been several studies on controlling the drug-release behaviour. Neohesperidin complexes with phospholipid/HP-β-cyclodextrin are more soluble and better absorbed in the gastrointestinal tract [49]. Cholesterol-based verbascoside phytosomes have been shown to increase vesicle rigidity, resulting in more controlled drug release [40]. These advances underscore the growing focus on engineering phytosome systems that deliver site-specific targeting and long-term therapeutic exposure.

### 9.3. Computationally Guided and Personalised Phytosome Design

The advent of computational technologies has created new possibilities for the rational formulation of the phytosomal system. The use of artificial intelligence, machine learning algorithms, molecular docking, and predictive modelling to elucidate drug-phospholipid interactions, optimise formulation variables, and identify an optimal drug-to-lipid ratio is becoming more common [60,125,126]. These methods will help reduce experimental workload and accelerate formulation development, enabling a more systematic design of next-generation phytosomal systems. At the same time, there is growing interest in personalised medicine, prompting research into patient-specific phytosomal products. The versatility of phytosome delivery systems in nutraceutical applications is demonstrated by the use of phytosome-enriched yoghurt containing O. abyssinica [27] and by the use of phytosome in multivitamin ice cream [72]. The use of advanced manufacturing technologies, such as 3D printing, has greater potential for personalised dosing and controlled drug release [127]. Such technologies can help develop individualised phytosomal therapies that address the needs and therapeutic requirements of individual patients.

These newer developments suggest that phytosome technology is advancing from a platform for bioavailability enhancement to a multifunctional drug delivery system, and is also being used to enable targeted delivery, controlled release, and personalised treatment strategies. The introduction of next-generation nanocarriers, surface engineering methods and computational formulation tools will facilitate the translation of phytosomal systems from conventional nutraceutical applications to precision medicine and the next generation of pharmaceutical products.

## 10. Regulatory Perspectives on Phytosomal Products

The advances in phytosome technology discussed above highlight continued development of these systems from formulation concepts toward products with potential clinical applications. However, progression from technological development to therapeutic products also depends on considerations beyond formulation performance, particularly the regulatory status of the final product and the evidence required for its intended use. In the United States, botanical products intended for therapeutic use are regulated as drugs, whereas products marketed as dietary supplements are regulated under a different framework; therefore, botanical drug development requires careful consideration of product quality, safety, and efficacy [122]. At the international level, the classification, requirements, and permitted uses of dietary supplements and herbal medicinal products vary between jurisdictions [123]. Consequently, the predominance of phytosomal products in the clinical literature as dietary supplements or adjunctive interventions does not necessarily indicate their development as prescription medicines, as these product categories differ in intended use, claims, and regulatory requirements.

The patent literature further illustrates the extent of technological development in this field. Phospholipid-complex technologies have been described for applications including cellulite, allergic and asthmatic conditions, skin and wound treatment, and other therapeutic or cosmetic uses []. More recently, an analysis of phytosome patents registered between 2000 and 2024 reported continued patent activity across pharmaceutical and nutraceutical applications [128]. These patents indicate technological interest in diverse phytosomal applications; however, patent protection does not by itself establish clinical efficacy, long-term safety, or regulatory approval.

This distinction is relevant when the patent landscape is considered alongside the clinical evidence identified in the present review. The patent literature encompasses a broader range of proposed applications than the ten human studies summarised in Table 2 [112,113,114,115,116,117,118,119,120,121], indicating that technological development has extended beyond the areas currently supported by clinical evidence. For pharmaceutical development, appropriate control of the botanical starting material, formulation composition, and manufacturing process is also important, as differences in botanical source and processing can affect chemical composition and reproducibility [129]. Thus, patents and commercial availability provide evidence of technological and market interest but should not be considered substitutes for clinical or regulatory evidence. The current gap between the breadth of patented applications and the available clinical evidence highlights the need for continued evaluation of phytosomal products within appropriate quality, clinical, and regulatory frameworks. These considerations also provide a basis for examining the broader challenges and opportunities that may influence the future development of phytosomal drug-delivery systems.

## 11. Challenges and Opportunities in Phytosomal Drug Delivery

The regulatory and evidentiary considerations discussed above are closely linked to the practical challenges involved in developing reproducible phytosomal products. The inherent variability of botanical materials and natural bioactives, arising from differences in source, processing, and storage, can complicate their characterisation, standardisation, and the reproducibility of formulation [122,130]. Appropriate control of the botanical starting material, phytochemical composition, formulation variables, and manufacturing process is therefore important for consistent product quality. Safety assessment is also an important consideration for products containing complex botanical matrices, particularly regarding adverse reactions, potential herb–drug interactions, and appropriate safety monitoring [131].

Manufacturing and dosage-form development present additional considerations. Scale-up requires consistent control of critical quality attributes, including particle size, polydispersity index, encapsulation efficiency, and stability, as well as batch-to-batch reproducibility. The conversion of phytosomal complexes into solid dosage forms also requires attention to processing and storage conditions. Spray drying and lyophilisation have been used to obtain dry phytosomal complexes [38,39], while recent work has explored phytosomal tablets using 3D printing [126]. However, the effects of drying, moisture exposure, phospholipid stability, and subsequent mechanical processing on phytosome integrity and drug-release behaviour require further evaluation.

Although preclinical phytosomal research has expanded considerably, the available clinical evidence remains limited and heterogeneous, as discussed in Section 8. Differences in formulation composition, study design, treatment duration, and clinical endpoints also limit direct comparison across studies. More broadly, the development of natural products for pharmaceutical applications involves challenges in characterisation, optimisation, standardisation, and development, all of which require coordinated analytical and formulation approaches [130]. Extending phospholipid complexation to less-explored natural bioactives from marine organisms, microorganisms, fungi, and algae may broaden the scope of phytosomal delivery. However, their formulation, safety, and pharmaceutical potential require further evaluation [129,130]. Addressing these formulation, manufacturing, stability, safety, and evidence gaps will help define the feasibility and broader applicability of phytosomal systems for pharmaceutical development.

## 12. Conclusions

Phytosomal delivery is fundamentally based on molecular complexation between natural bioactives and phospholipids, with phospholipid–membrane interactions contributing to their delivery behaviour. Formulation composition and preparation conditions influence critical quality attributes and, consequently, the physicochemical, release, and pharmacokinetic characteristics of the resulting systems. This provides a common design framework that links molecular interactions to formulation behaviour and delivery performance. Emerging approaches, including hybrid systems, targeted delivery, alternative routes of administration, and computational formulation, provide opportunities to tailor phytosomal delivery further. However, the available clinical evidence remains limited and heterogeneous, and improvements in formulation or pharmacokinetic performance should not be equated with established clinical efficacy. The predominance of phytosomal products as dietary supplements or adjunctive interventions also highlights the distinction between formulation development and medicinal product development, emphasising the need for appropriate assessments of quality, safety, and efficacy, as well as regulatory evaluation. Future studies should focus on reproducible complexation, standardised formulation and manufacturing approaches, and robust pharmacokinetic and clinical evaluation. Extending phospholipid complexation to less-explored natural bioactives from marine, microbial, fungal, and algal sources may broaden the scope of phytosomal delivery, subject to adequate evaluation. However, future progress will depend on integrating advances in molecular complexation and formulation engineering with robust pharmacokinetic and clinical validation, while addressing standardisation, manufacturing, and regulatory challenges to support the broader development of phytosomal delivery systems.

## Figures and Tables

**Figure 1:**
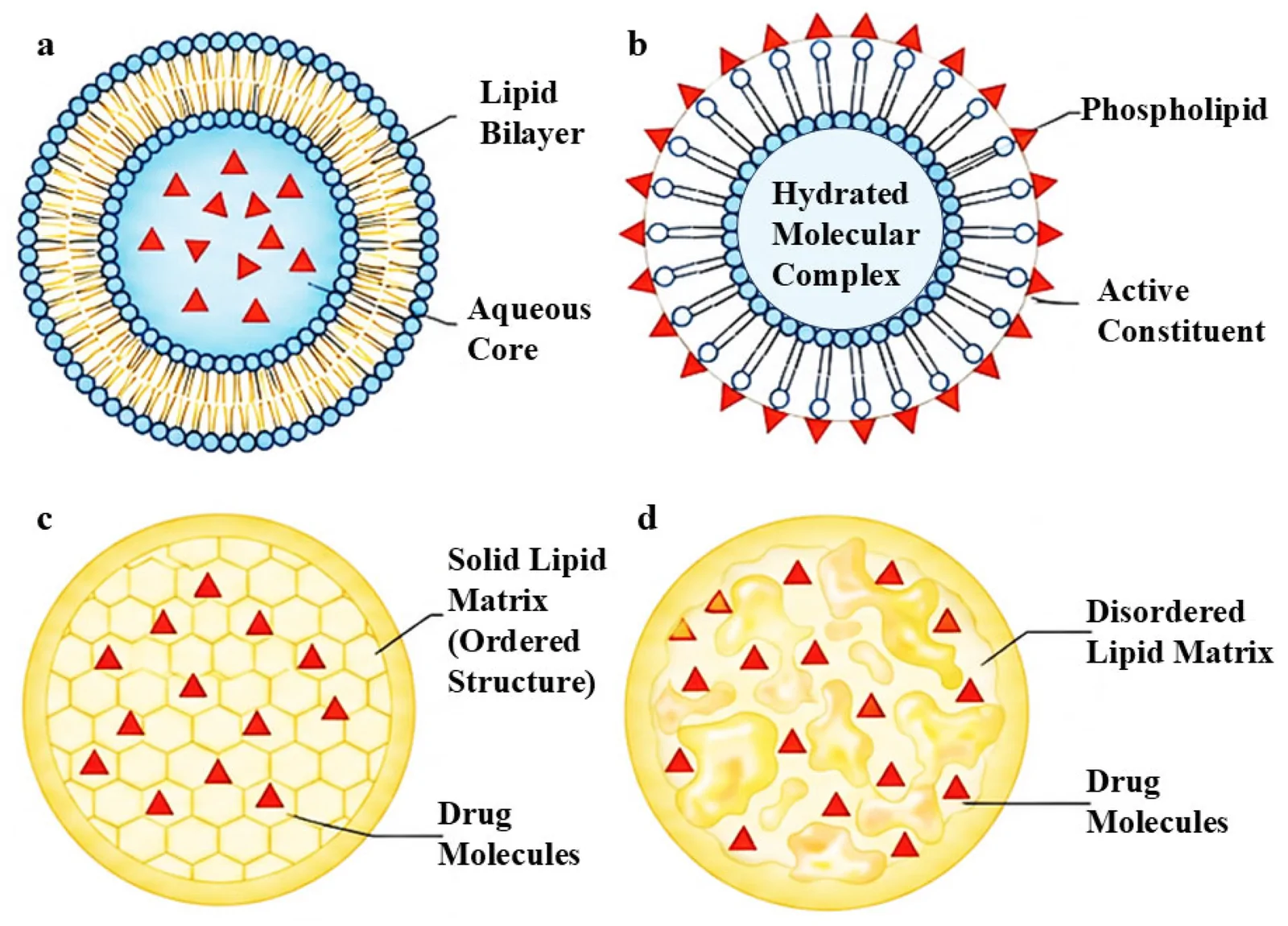
Comparative structural organisation and drug association mechanisms of (**a**) Liposome, (**b**) Phytosome, (**c**) Solid Lipid Nanoparticle (SLN), (**d**) Nanostructured Lipid Carrier (NLC).

**Figure 2:**
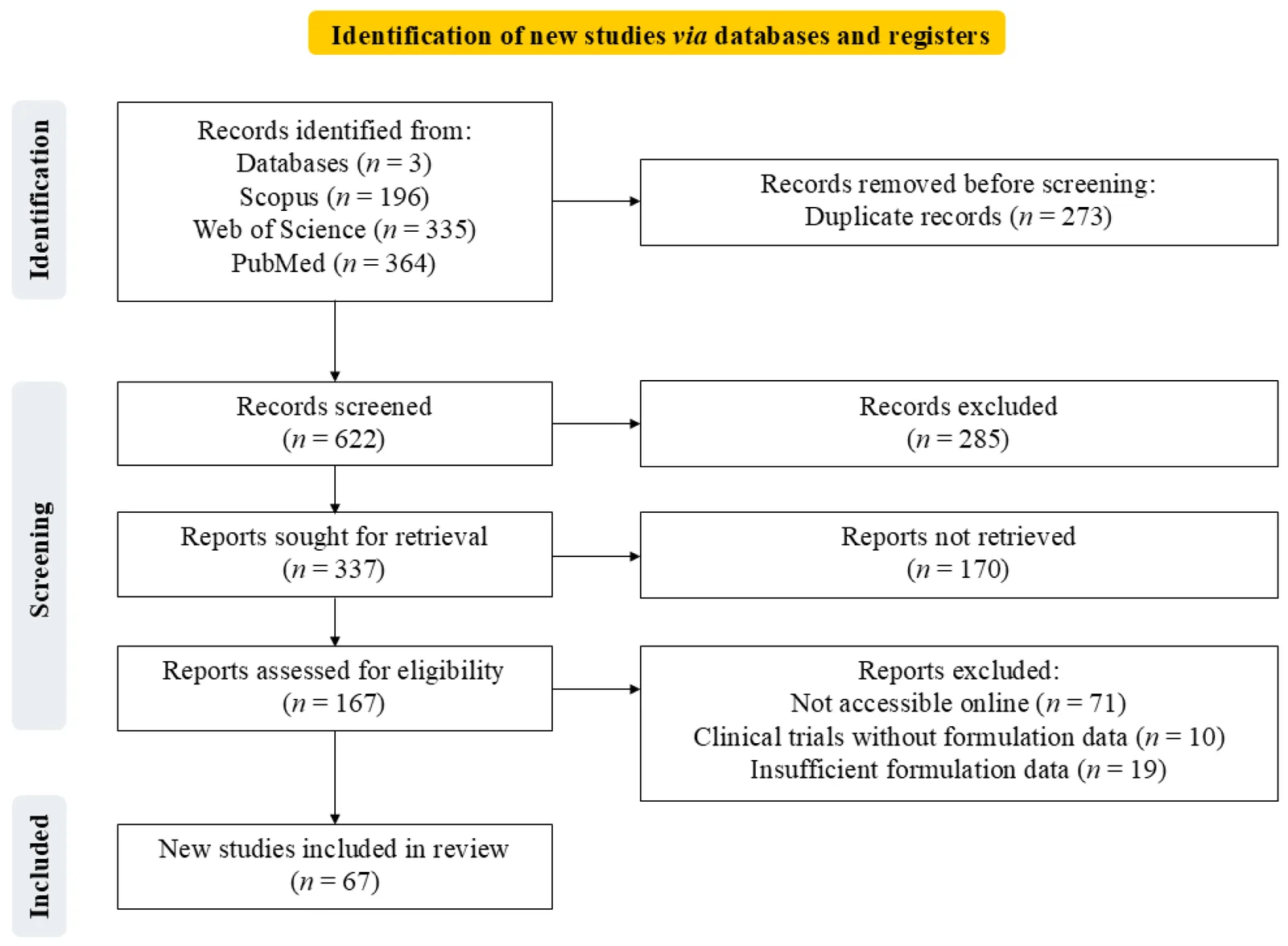
PRISMA 2020 flow diagram illustrating identification, screening, eligibility assessment, and inclusion of studies [10].

**Figure 3:**
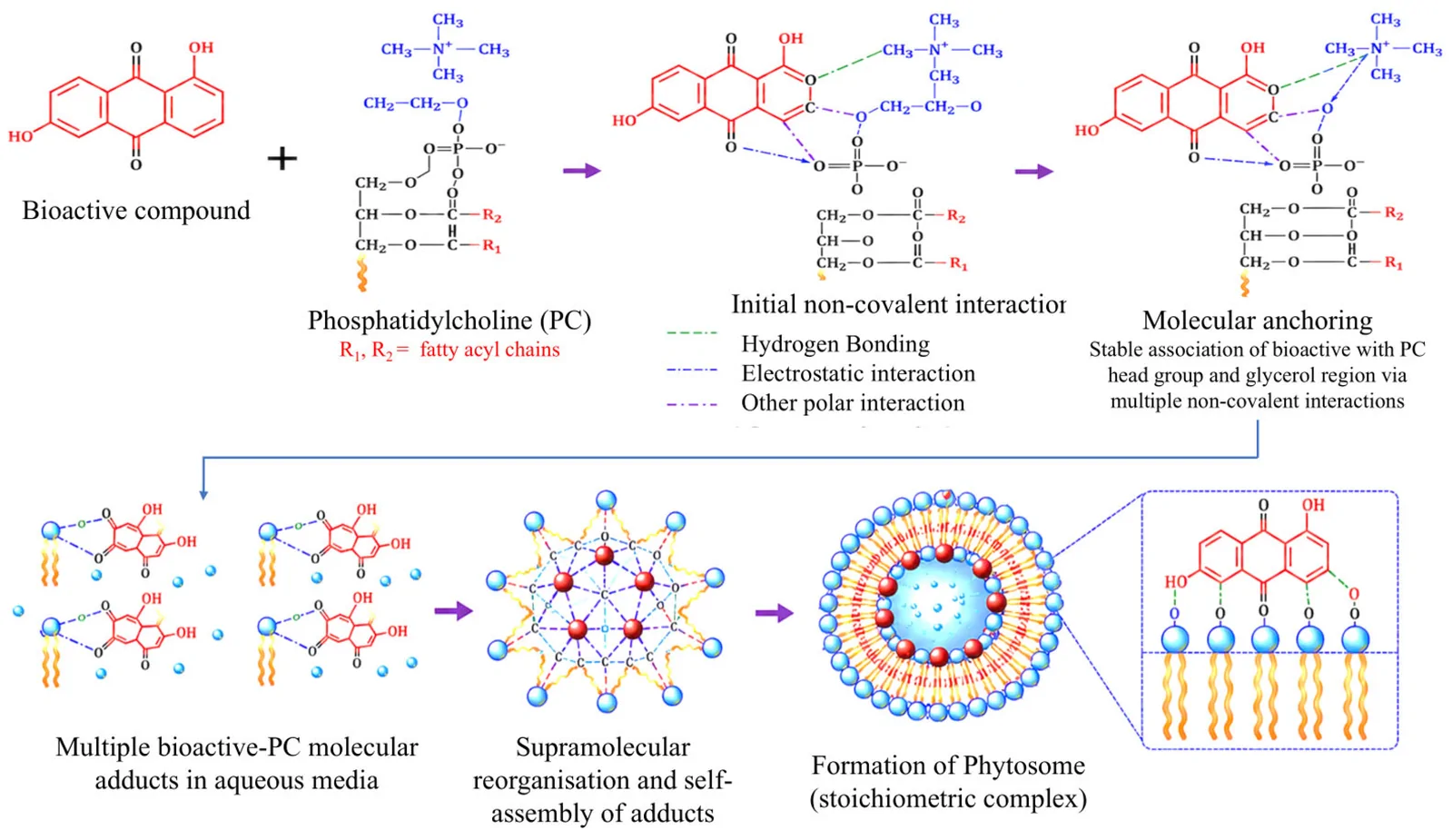
Schematic illustration of phytosome formation through non-covalent interactions between natural bioactive compounds and phosphatidylcholine, resulting in molecular anchoring, supramolecular reorganisation, and self-assembly into stable phytosomal complexes.

**Figure 4:**
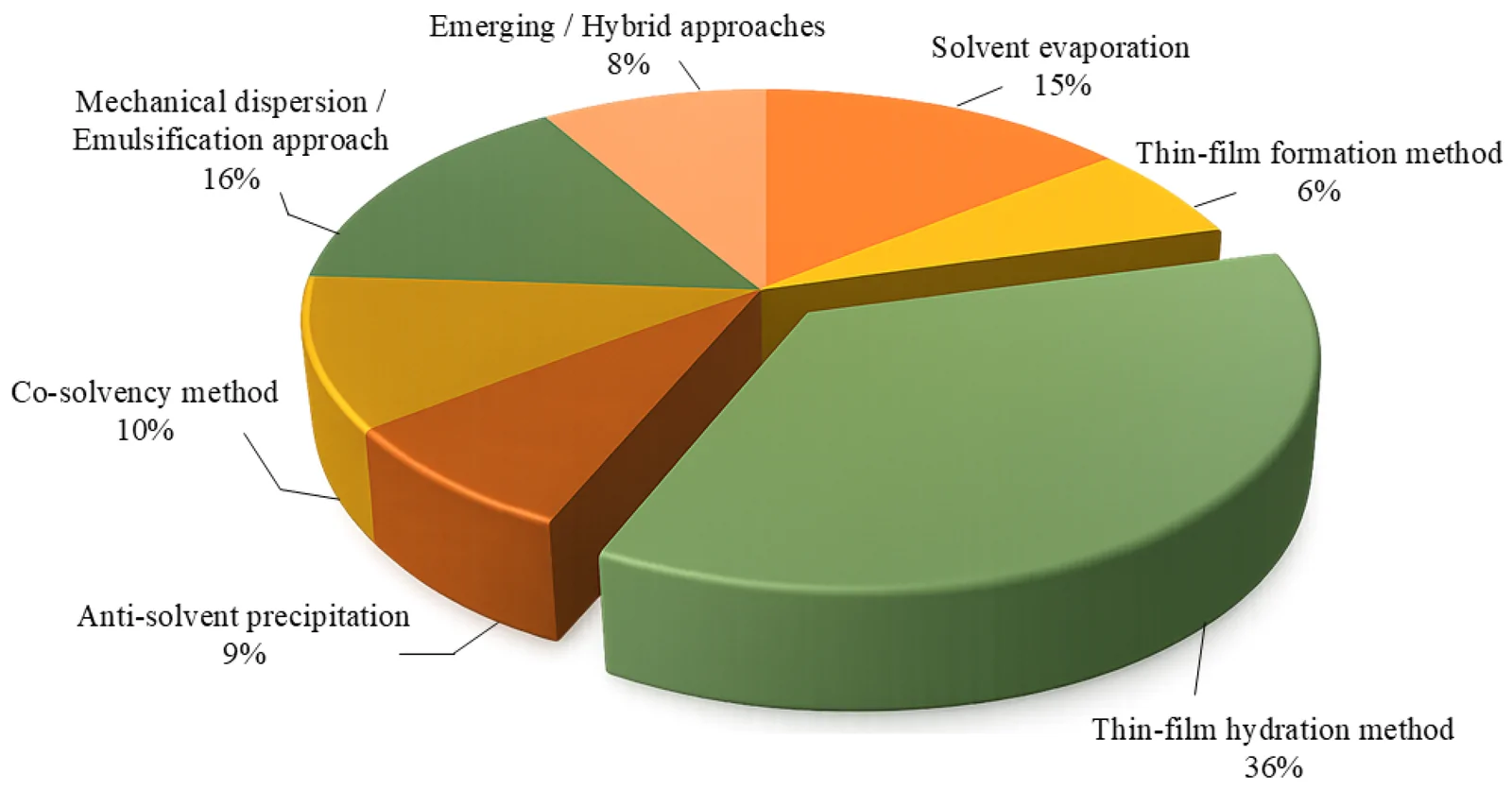
Representing the distribution of phytosome preparation methods reported in the literature included in this review.

**Figure 5:**
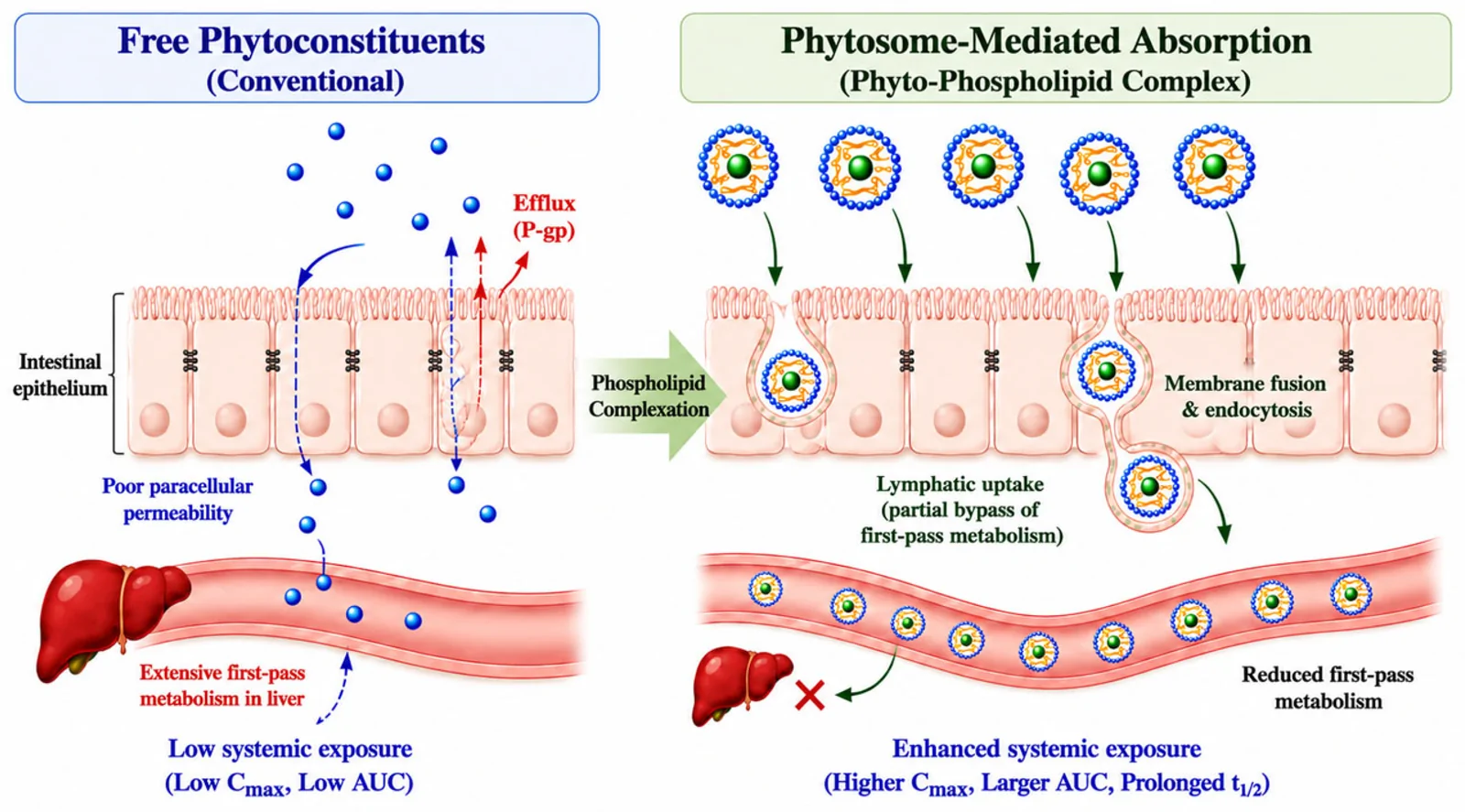
Schematic illustration of improved intestinal absorption, lymphatic transport, membrane interaction, and reduced first-pass metabolism following phytosome administration.

**Figure 6:**
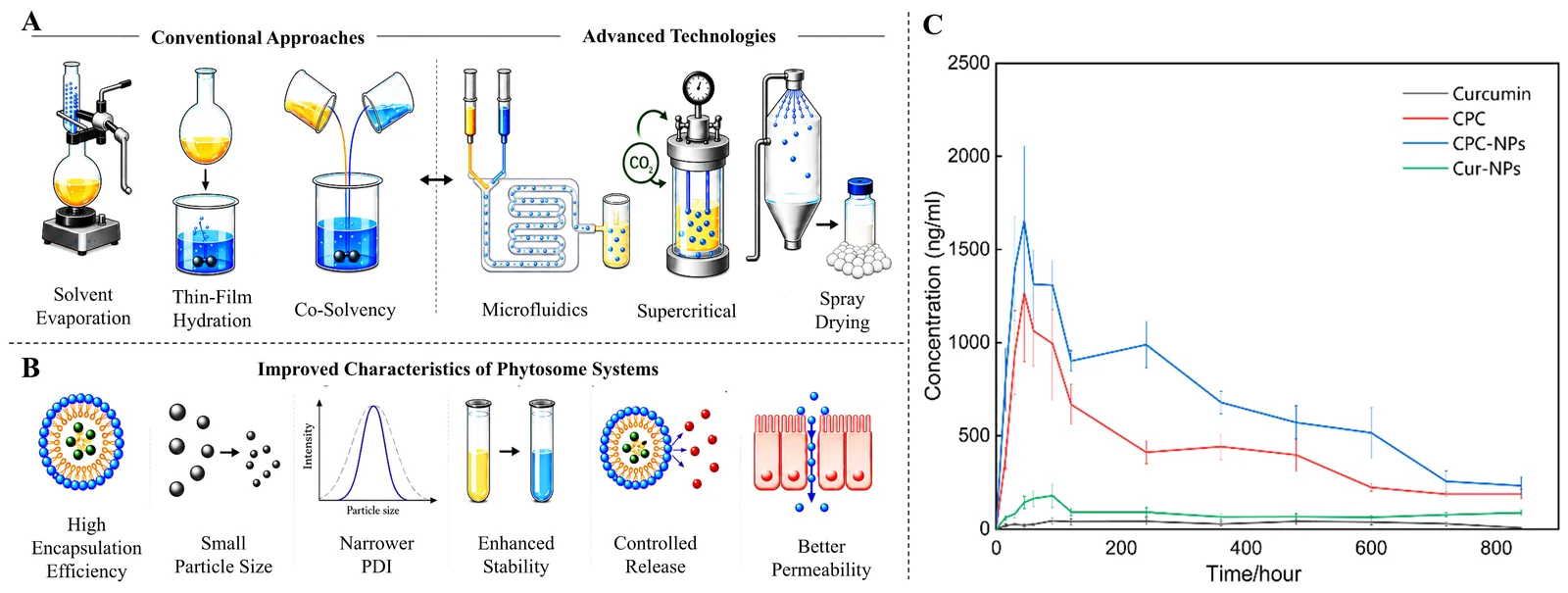
Evolution of phytosome preparation technologies and their influence on formulation performance and oral bioavailability. (**A**) Conventional and advanced phytosome manufacturing approaches, including solvent evaporation, thin-film hydration, co-solvency, microfluidics, supercritical CO_2_ processing, and spray drying/lyophilisation. (**B**) Physicochemical improvements associated with advanced phytosomal systems, including enhanced entrapment efficiency, reduced particle size, narrower polydispersity index (PDI), improved stability, controlled drug release, and enhanced permeability. (**C**) Representative plasma concentration-time profiles of free curcumin, curcumin–phospholipid complex (CPC), curcumin nanoparticles (Cur-NPs), and curcumin–phospholipid complex nanoparticles (CPC-NPs) following oral administration data in the panel were adapted from [92].

**Figure 7:**
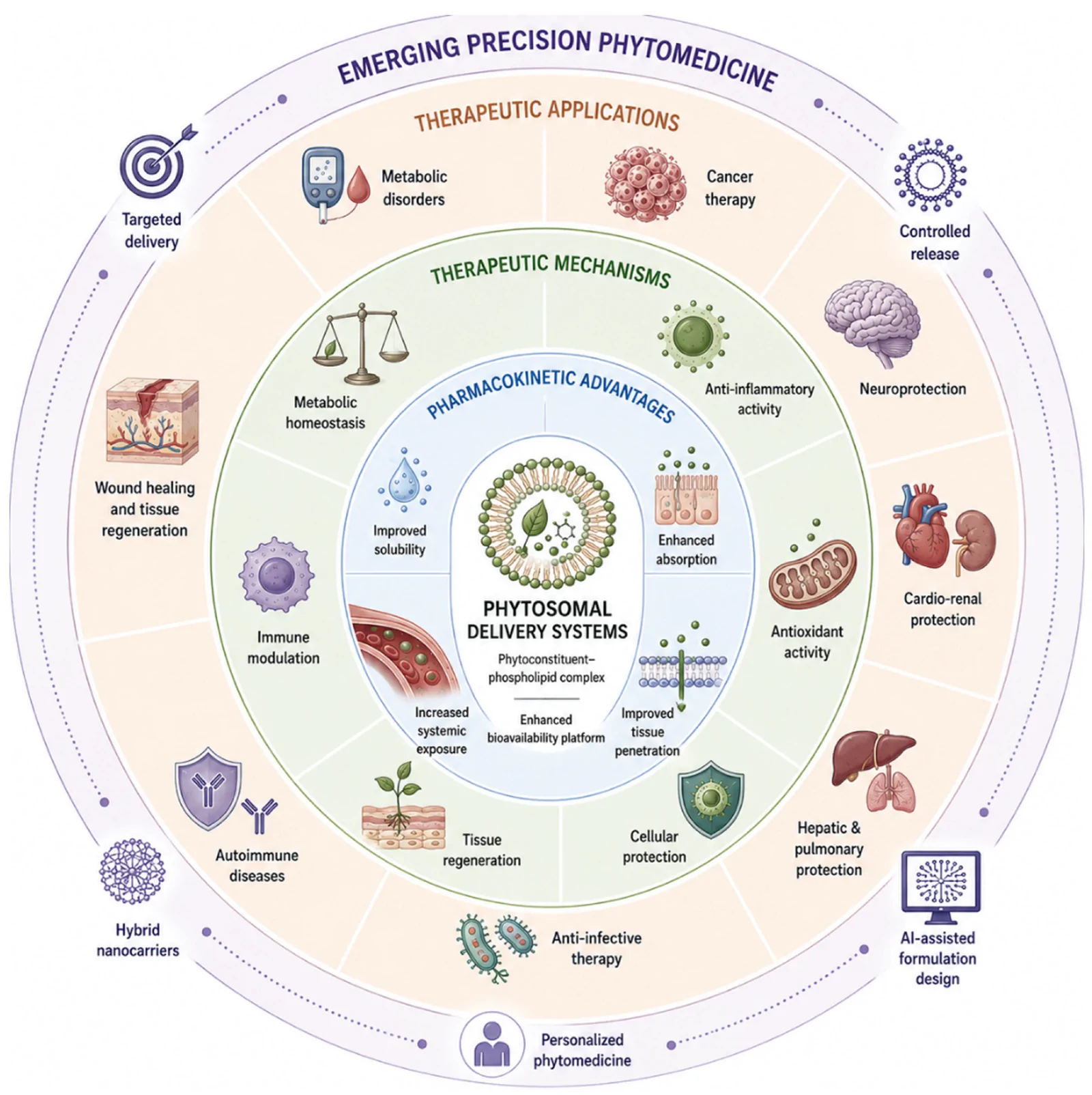
Schematic overview of the pharmacokinetic advantages, therapeutic mechanisms, clinical applications, and emerging precision medicine strategies associated with phytosomal delivery systems. Improved solubility, absorption, tissue penetration, systemic exposure, and bioavailability contribute to diverse biological activities and therapeutic applications. Emerging developments include targeted delivery, controlled release systems, hybrid nanocarriers, AI-assisted formulation optimisation, and personalised phytomedicine.

**Table 2:** Summary of recent clinical trials on phytosome-based delivery system.

Sl. No	Clinical Trial Registration Number	Disease/Clinical Condition	Population and Study Design	Intervention Dose & Dosage Form	Duration of Intervention	Primary Outcome	Key Clinical Findings	Safety/Tolerability	Reference
1	NCT05480670	Polycystic ovary syndrome (PCOS)	130 women aged between 18 and 45 years were involved in a controlled, randomised, multi-centred, and open-label clinical trial	berberine phytosome (550 mg × 2/die) was given orally	Ninety days	Resumption of regular menstruation (*p* < 0.0001), normalisation of the ovarian anatomy (*p* < 0.0001), and acne improvement (*p* = 0.0409), Hirsutism reduction (*p* = 0.0152) has been observed	A study showed the positive effects of berberine phytosome supplementation in women with PCOS compared to the control group	Well tolerated, and no adverse reaction was reported	[112]
2	NCT05602688	Emotional distress and/or poor sleep conditions	100 healthy adults were involved in a randomised, placebo-controlled, parallel-group, double-blinded clinical trial	phospholipid carrier-based *Melissa officinalis* L. leaf extract (200 mg/die) was given orally	Three weeks	Improvements in the depressive mood, anxiety, stress, positive and negative affect (emotional feelings), overall mental well-being, and quality-of-life scores (all *p* values < 0.001).	Promising therapeutic option for the management of moderate emotional distress and/or poor sleep conditions	Well tolerated; no adverse reactions were reported	[113]
3	NCT05730660	Chronic fatigue	42 females and 36 males, in total 78 subjects of mean age 56 ± 9, were involved in a double-blind, placebo-controlled clinical trial	lecithin-based delivery form of quercetin (Quercetin phytosome) 500 mg/day was given orally	Two-month	significant changes between groups have been recorded in the Pittsburgh Sleep Quality Index, physical performance, and FIS-40 mean (all *p*-values < 0.001).	The quercetin supplementation counterbalances Chronic fatigue symptoms.	-	[114]
4	NCT05730998	Urinary tract infections	46 diabetic postmenopausal female subjects aged ≥70 were involved in a randomised, double-anonymised, placebo-controlled, parallel-group, six-month clinical supplementation study	cranberry extract phytosome (Anthocran™ phytosome) 120 mg/day was given orally	Six months	9% of the placebo group showed infection, whereas only 3% of those with cranberry supplementation did. Glycaemia and glycated haemoglobin values were not modified	Highly standardised cranberry extract phytosome supplementation reduced UTI recurrence	Well tolerated, and no adverse reaction was reported	[115]
5	-	Cardiovascular risk with dyslipidaemia	60 consecutive patients of mean age 48.02 ± 10.1 years were involved in a prospective single-arm observational study	leucoselect^®^ phytosome^®^ (300 mg), red yeast rice extract (99 mg), monacolin K (2.97 mg), polycosanols (10 mg), octacosanol (9 mg), folic acid (200 mcg) was given once daily	Twelve weeks	Significant reduction in phospholipaseA2(PLA2) (*p* = 0.001)	Significant reduction of 20% in LDL-C serum level in patients with dyslipidaemia at low cardiovascular risk	Well tolerated, and no adverse reaction was reported	[116]
6	NCT05950932	Mild anxiety and insomnia	30 participants (13 males and 17 females) aged 18 to 65 years, were involved in a prospective, double-blind, placebo-controlled, crossover, and non-profit study	*Melissa officinalis* phytosome (200 mg/day) given orally	One month	Significant reduction in the ISI score (*p* = 0.001), with 87% of participants in the treated group reporting improved sleep quality	Showed significant improvements in insomnia severity index scores and an increase in deep sleep	Well tolerated, and no adverse reaction was reported.	[117]
7	NCT06391606	Statin-associated asthenia	60 participants (33 males and 27 females) aged 65–80 years were involved in a randomised, double-anonymised, placebo-controlled clinical study	Coenzyme Q10 phytosome (Ubiqsome^®^ 300 mg/daily) given orally	Eight weeks	Greater improvement in asthenia, HGS, 2MST, and 1 min STS repetitions compared to the placebo group (*p* < 0.05)	Effectively improved physical performance in older adults with statin-associated asthenia.	Well tolerated; no adverse reactions were reported.	[118]
8	-	Irritable bowel syndrome (IBS)	49 participants (37 females and 12 males) with a mean (±SD) age of 43.9 (±14.4) years, were involved in a randomised, double-blinded, placebo-controlled, parallel-group study	Lecithin-based delivery formulation of *Curcuma longa* (250 mg) and *Boswellia serrata* (125 mg) twice a day, given orally	Thirty days	Significant reduction in urinary indican, abdominal bloating, and abdominal pain (all *p*-value < 0.001)	Has demonstrated a significant ability to improve gut health in subjects with IBS. When used in conjunction with a low-FODMAP diet, it reduces intestinal dysbiosis in subjects with IBS.	Well tolerated; no adverse reactions were reported.	[119]
9	NCT06652035	Hyperuricemia	250 participants (128 females and 122 males) were involved in a retrospective, prospective, cross-sectional, observational, multi-cohort study	Quercetin phytosome (Quevir^®^ 500 mg)	Ninety days	reduction in serum uric acid and triglyceride levels (*p* < 0.01)	Exhibits a significant hypouricemic effect in healthy adults with various metabolic conditions, as well as notable triglyceride-lowering properties.	Well tolerated; No adverse reactions were reported.	[120]
10	-	Seasonal allergic rhinitis (SAR)	Thirty children (14 females and 16 males) with a mean age of 10.7 years were involved in a case study using an observational, retrospective, controlled design.	Quercimmun™ Phytosome^®^ (200 mg), which contains quercetin (80 mg), zinc (3.5 mg), and vitamin C (20 mg). Two sticks/daily supplemented to cetirizine; 1 drop/3 kg/bw	Two months	Total nasal symptoms score significantly reduced compared to the control (*p* < 0.05)	An add-on strategy significantly reduces SAR symptom severity, also after discontinuation of antihistaminic therapy.	Well tolerated, with no adverse reactions reported.	[121]

PCOS, polycystic ovary syndrome; UTI, urinary tract infection; IBS, irritable bowel syndrome; SAR, seasonal allergic rhinitis; LDL-C, low-density lipoprotein cholesterol; PLA2, phospholipase A2; ISI, insomnia severity index; FIS-40, fatigue impact scale-40; HGS, handgrip strength; 2MST, 2 min step test; 1 min sts, 1 min sit-to-stand test. Most phytosome formulations were administered orally either as dietary supplements or adjunct therapies. “-” indicates information not reported. The table summarises the principal efficacy and safety outcomes of published human clinical studies on phytosome-based formulations.

## Data Availability

The data supporting the review’s findings can be found in the cited literature.

## Terminology and Abbreviations

Terminology: In this review, “phytosome” is used as the general term for phospholipid-based complexes of natural bioactives; “phospholipid complex” and “phyto-phospholipid complex” are retained when reflecting the terminology of the cited studies or referring specifically to the complexation process; “nanophytosome” is reserved for systems explicitly described as nanoscale phytosomes. The following abbreviations are used in this manuscript:

AUC	Area under the curve
Cmax	Maximum plasma concentration
CQA	Critical quality attribute
Tmax	Time to maximum plasma concentration
PK	Pharmacokinetics
PD	Pharmacodynamics
EE	Entrapment efficiency
DL	Drug loading
PDI	Polydispersity index
ZP	Zeta potential
QbD	Quality by design
GMP	Good manufacturing practice
TEM	Transmission electron microscopy
SEM	Scanning electron microscopy
NLC	Nano-lipid carrier
SLN	Solid lipid nanocarrier
PC	Phosphatidyl choline
RCT	Randomised clinical trial
BCS	Biopharmaceutical classification system
